# Author Correction: Dynamic relocalization of cytosolic type III secretion system components prevents premature protein secretion at low external pH

**DOI:** 10.1038/s41467-021-23080-5

**Published:** 2021-05-05

**Authors:** Stephan Wimmi, Alexander Balinovic, Hannah Jeckel, Lisa Selinger, Dimitrios Lampaki, Emma Eisemann, Ina Meuskens, Dirk Linke, Knut Drescher, Ulrike Endesfelder, Andreas Diepold

**Affiliations:** 1 Department of Ecophysiology, Max Planck Institute for Terrestrial Microbiology, Marburg, Germany; 2 Max Planck Institute for Terrestrial Microbiology, Marburg, Germany; 3 Department of Physics, Philipps-Universität Marburg, Marburg, Germany; 4 Department of Biosciences, University of Oslo, Oslo, Norway; 5 SYNMIKRO, LOEWE Center for Synthetic Microbiology, Marburg, Germany; 6 Present Address: Department of Physics, Mellon College of Science, Carnegie Mellon University, Pittsburgh, PA USA; 7 Present Address: Max-Planck-Institut für Immunbiologie und Epigenetik, Freiburg, Germany; 8 Present Address: James Madison University, Harrisonburg, VA USA

**Keywords:** Bacterial pathogenesis, Bacterial secretion, Pathogens

Correction to: *Nature Communications* 10.1038/s41467-021-21863-4, published online 12 March 2021.

The original version of this Article contained an error in the Methods, section ‘Single-particle tracking photoactivated localization microscopy (sptPALM)’, where an equation incorrectly read: D = M^2^π^−1^−σ_m_^2^τ^−1^ . The correct form of the equation is: D = (M^2^π^−1^−σ_m_^2^)τ^−1^.

This has been corrected in both the PDF and HTML versions of the Article.

