# Supplementary material for: Dynamic relocalization of cytosolic type III secretion system components prevents premature protein secretion at low external pH
Source: Nat Commun. 2021 Mar 12;12:1625. doi: 10.1038/s41467-021-21863-4 (PMC7954860; doi:10.1038/s41467-021-21863-4)
Supplement: Supplementary file 1 — Supplementary Information [file 41467_2021_21863_MOESM1_ESM.pdf]

<u>Supplementary information for</u>

Dynamic relocalization of cytosolic type III secretion system components prevents premature protein secretion at low external pH

Stephan Wimmi[1], Alexander Balinovic[2], Hannah Jeckel[2,3], Lisa Selinger[1], Dimitrios Lampaki[1*], Emma Eisemann[1*], Ina Meuskens[4], Dirk Linke[4], Knut Drescher[2,3], Ulrike Endesfelder[2*] & Andreas Diepold[1,5]*

1: Max Planck Institute for Terrestrial Microbiology, Department of Ecophysiology, Karl-von-Frisch-Str. 10, 35043 Marburg, Germany

2: Max Planck Institute for Terrestrial Microbiology, Karl-von-Frisch-Str. 10, 35043 Marburg, Germany

3: Department of Physics, Philipps-Universität Marburg, Karl-von-Frisch-Str. 16, 35043 Marburg, Germany

4: University of Oslo, Department of Biosciences, Blindernveien 31, 0371 Oslo, Norway

5: SYNMIKRO, LOEWE Center for Synthetic Microbiology, Marburg, Germany

* current addresses:
Alexander Balinovic, Department of Physics, Mellon College of Science, Carnegie Mellon University, 5000 Forbes Avenue, Pittsburgh, PA 15213, USA;
Dimitrios Lampaki, Max-Planck-Institut für Immunbiologie und Epigenetik, Stübeweg 51, 79160 Freiburg, Germany;
Emma Eisemann, James Madison University, 800 South Main St, Harrisonburg, VA 22807, USA;
Ulrike Endesfelder, Department of Physics, Mellon College of Science, Carnegie Mellon University, 5000 Forbes Avenue, Pittsburgh, PA 15213, USA

Correspondence: Andreas Diepold, andreas.diepold@mpi-marburg.mpg.de, +49-6421-178302

Supplementary Figures

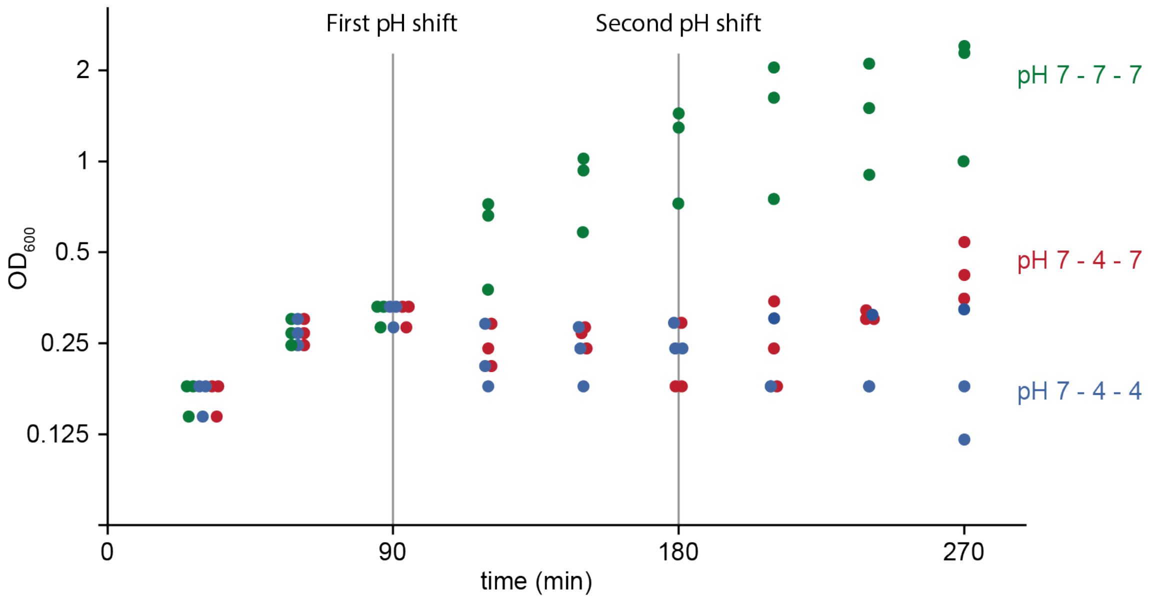

Supplementary Figure 1 – *Y. enterocolitica* growth stops at pH 4, and can be resumed in neutral pH

Y. enterocolitica were inoculated to an OD_{600} of 0.12 from a stationary overnight culture. They were grown at pH 7 (28°C) for 90 min (0-90 min), collected by centrifugation and then resuspended in fresh medium (37°C) at pH 7 (green data points) or pH 4 (red and blue data points). Cultures were incubated for another 90 min (90-180 min), again collected by centrifugation, resuspended in fresh medium (37°C) at pH 7 (green and red data points) or pH 4 (blue data points), and incubated for further 90 min (180-270 min). OD_{600} values were recorded every 30 min. The results of three independent experiments are displayed.

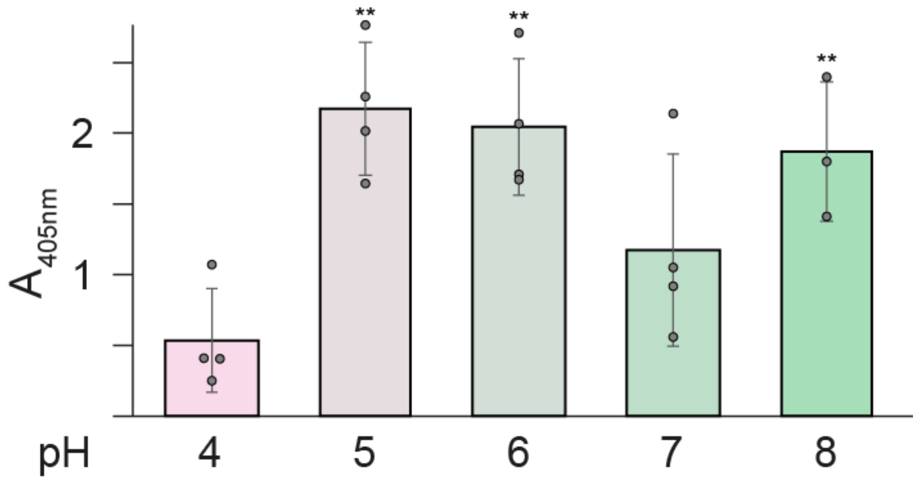

Supplementary Figure 2 – Binding of the *Y. enterocolitica* adhesin YadA to collagen at the indicated pH values.

Absorption at 405 nm resulting from Ni^{2+}-HRP binding to YadA-His_6 incubated with plate-absorbed calf collagen type I. n=4 independent biological replicates (n=3 for pH 8) with 3-8 technical replicates each. Bars denote mean values of the biological replicates, error bars denote standard deviation. Single data points indicated by small circles. **, statistically significant different compared to pH 4, $0.01>p>0.001$ in a homoscedastic two-tailed t-test, all other pairwise comparisons do not differ in a statistically significant way (p~0.001, 0.002, 0.15, 0.009 for comparisons between pH 4 and pH 5-8, respectively). The lower and less coherent adhesion at pH 7 might be due to precipitation of YadA close to its isoelectric point (pI_{YadA} = 7.2).

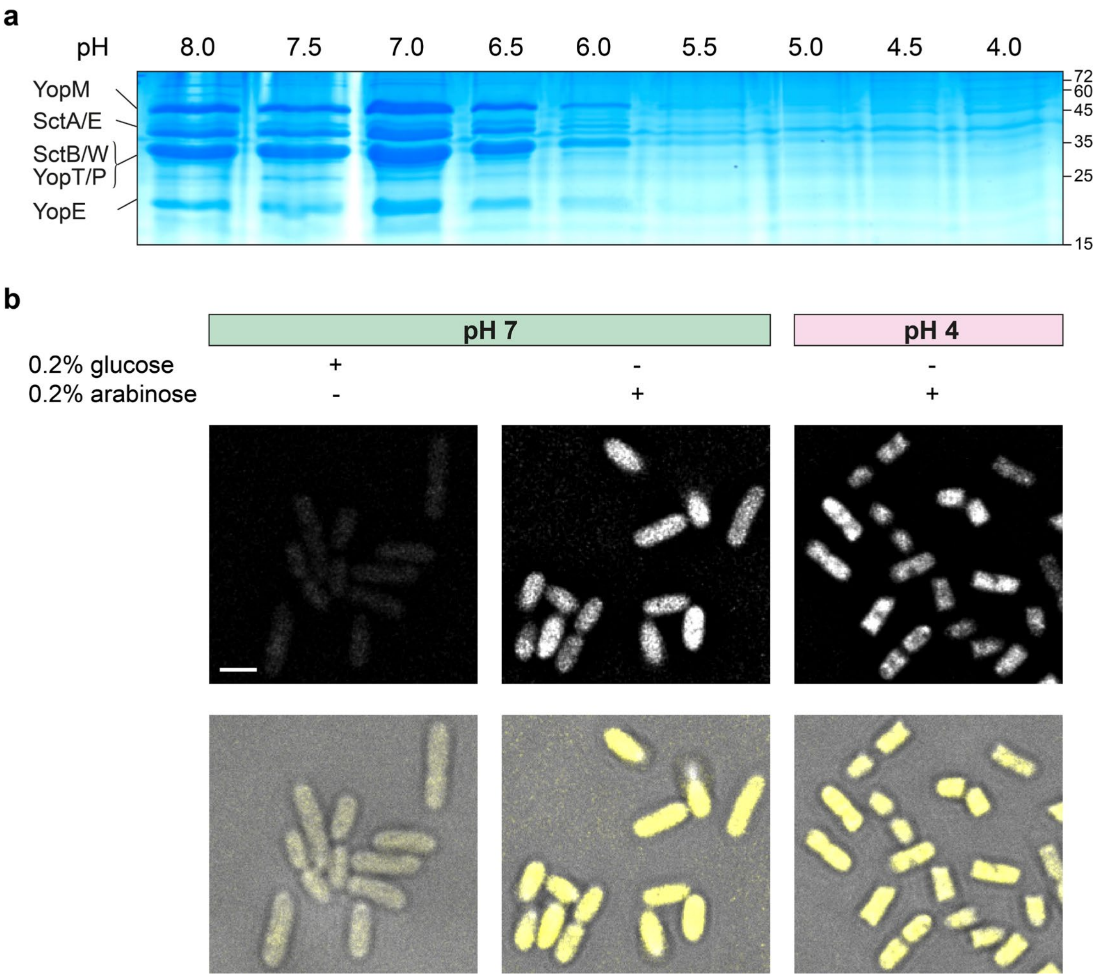

Supplementary Figure 3 – Protein export, but not protein synthesis in *Y. enterocolitica* is suppressed at an external pH of 4

(**a**) *In vitro* secretion assay showing the export of native T3SS substrates (indicated on left side) in a MRS40-based strain containing all native virulence effectors at the indicated external pH values. Coomassie-stained SDS-PAGE gel; supernatant of $3*10^8$ bacteria per lane. Right side, molecular weight marker (kDa). *n*>5 independent experiments. (**b**) *Y. enterocolitica* IML421asd (ΔHOPEMTasd) were grown at neutral pH and then subjected to different pH as indicated. EGFP expression was induced from a pBAD plasmid at the same time, and fluorescence was determined after 180 min. Top, fluorescence image in GFP channel, bottom, overlay of phase contrast (grey) and fluorescence (yellow). *n*=3 independent experiments. Scale bar, 2 µm.

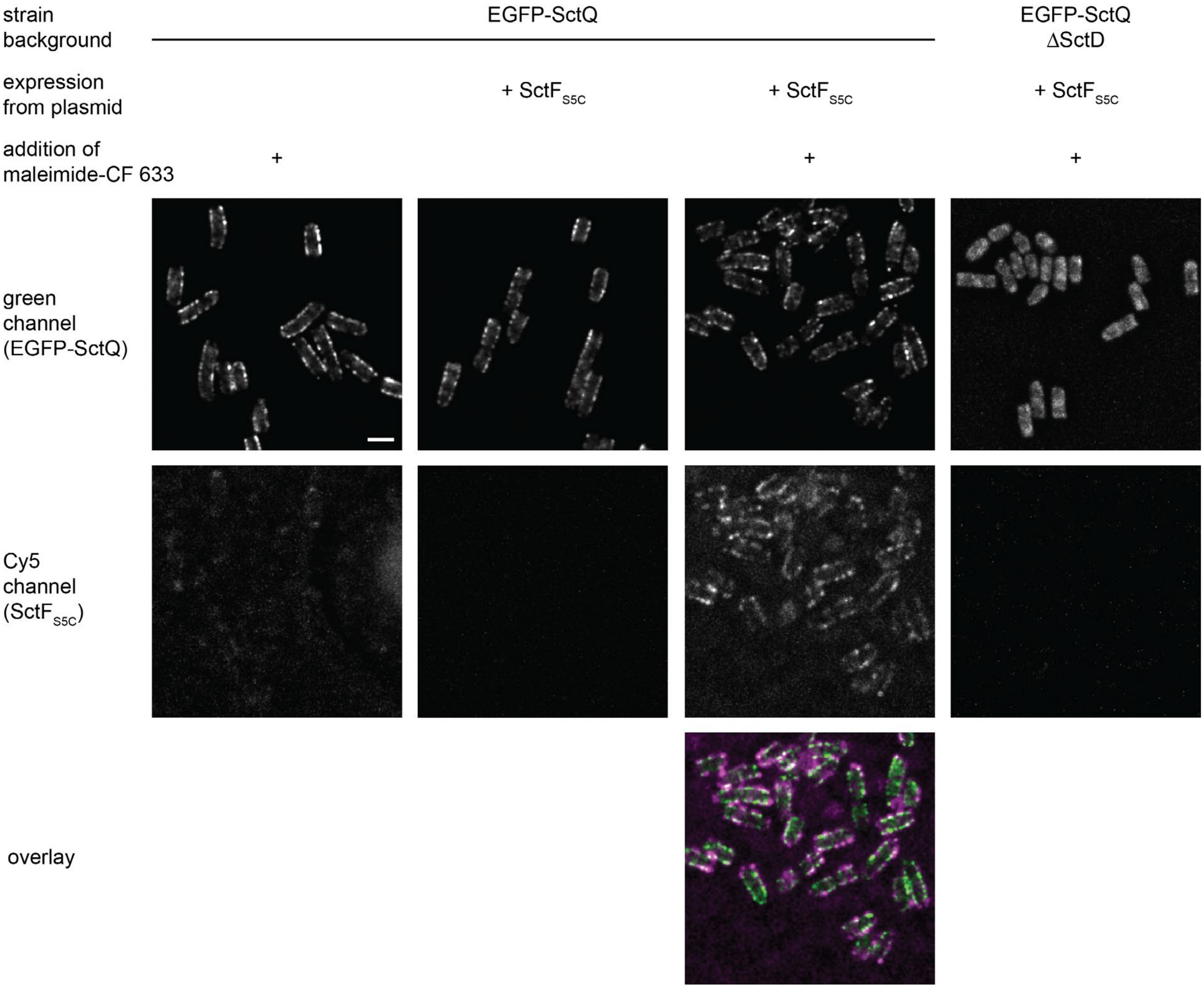

Supplementary Figure 4 – Specificity of maleimide-based labeling of SctF$_{S5C}$

Fluorescence micrographs of *Y. enterocolitica* strains expressing EGFP-SctQ from the native locus and SctF$_{C5S}$ from plasmid, where indicated, induced with 1.0% arabinose. Bacteria were stained using CF 633 maleimide dye, where indicated, and imaged in the green and Cy5 channel (shown in magenta in the overlay). *n*=3 independent experiments. Scale bar, 2 µm.

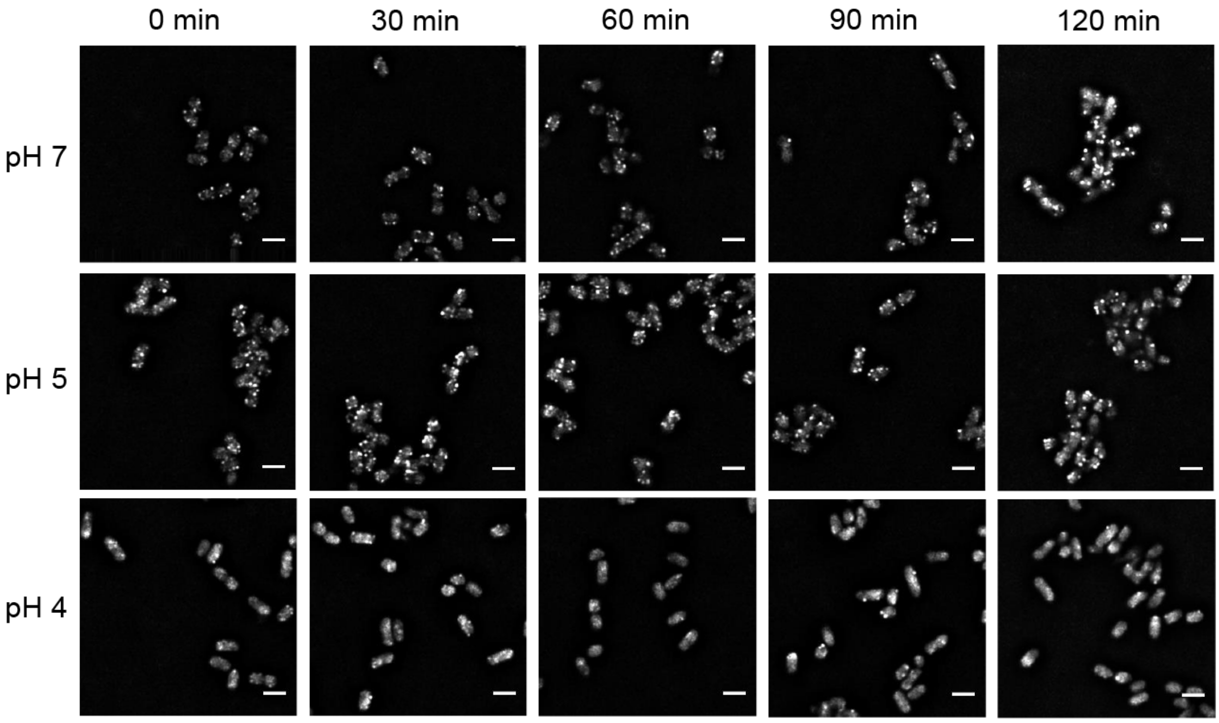

Supplementary Figure 5 – The localization of the cytosolic components remains stable over time

Fluorescence micrographs of *Y. enterocolitica* EGFP-SctQ, incubated at the indicated external pH values under secreting conditions over time. *n*=2 independent experiments. Scale bars, 2 µm.

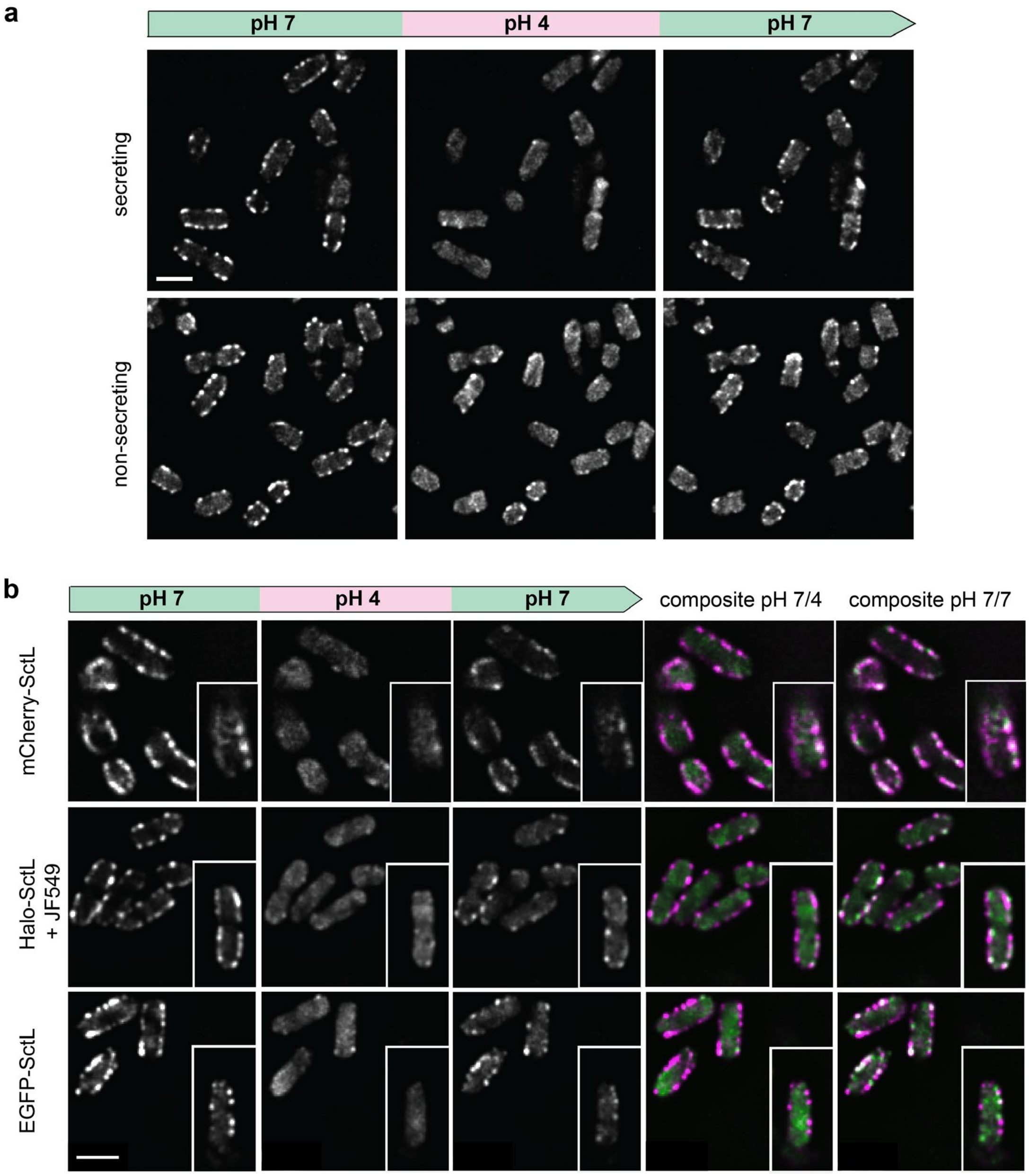

Supplementary Figure 6 – Dissociation of the cytosolic components at low external pH can be observed irrespective of the used visualization tag, and in both secreting and non-secreting conditions

(**a**) Fluorescence micrographs of *Y. enterocolitica* EGFP-SctQ, incubated at the indicated external pH values under secreting conditions (top), or non-secreting conditions (bottom) over time. (**b**) Fluorescence micrographs of *Y. enterocolitica* expressing indicated labeled versions of SctL (replacing the WT gene by allelic exchange) at the indicated external pH values under secreting conditions over time. Right columns, composite images; magenta: pH 7 (first image on the left); green: pH 4 (second image on the left) or pH 7 (third image on the left), as indicated. *n*=3 independent experiments. Scale bars, 2 µm.

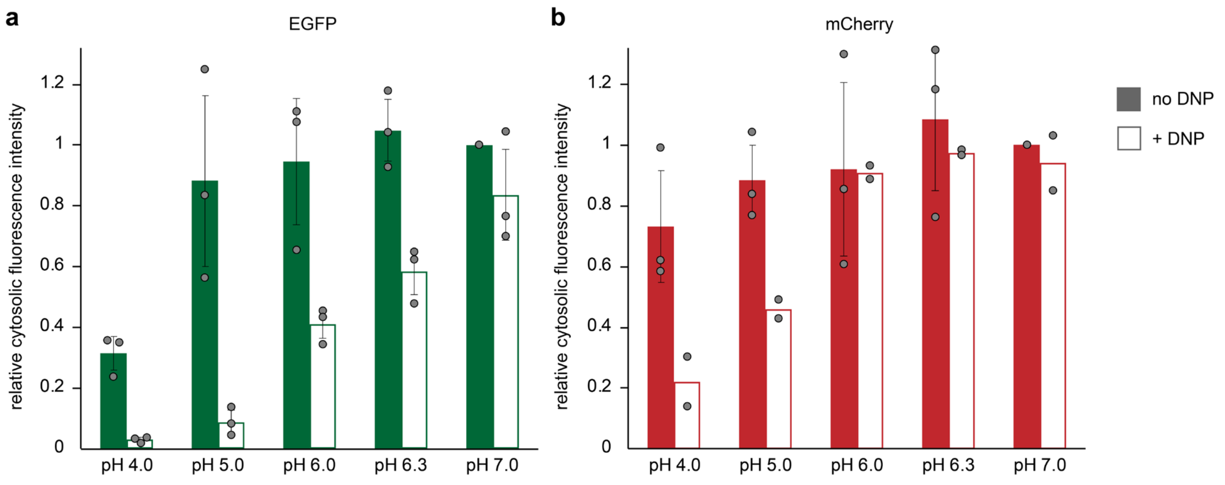

Supplementary Figure 7 – Intensity of cytosolic fluorescence levels of EGFP and mCherry at different external pH

The cellular fluorescence levels of cytosolic EGFP (**a**) and mCherry (**b**) were determined at the indicated external pH, in absence (filled bars) and presence (empty bars) of the ionophore 2,4-dinitrophenol (DNP, 2 mM), which adjusts the cytosolic pH to the external pH. Fluorescence per bacterium was determined by dividing the measured fluorescence above background by the number of bacteria per field of view, and normalized by the respective value at pH 7 in the absence of DNP. Error bars display the standard deviation of 3 independent experiments (2 independent experiments for mCherry + DNP). Bars denote mean values, single data points indicated by small circles. For each experiment, two fields of view were analyzed.

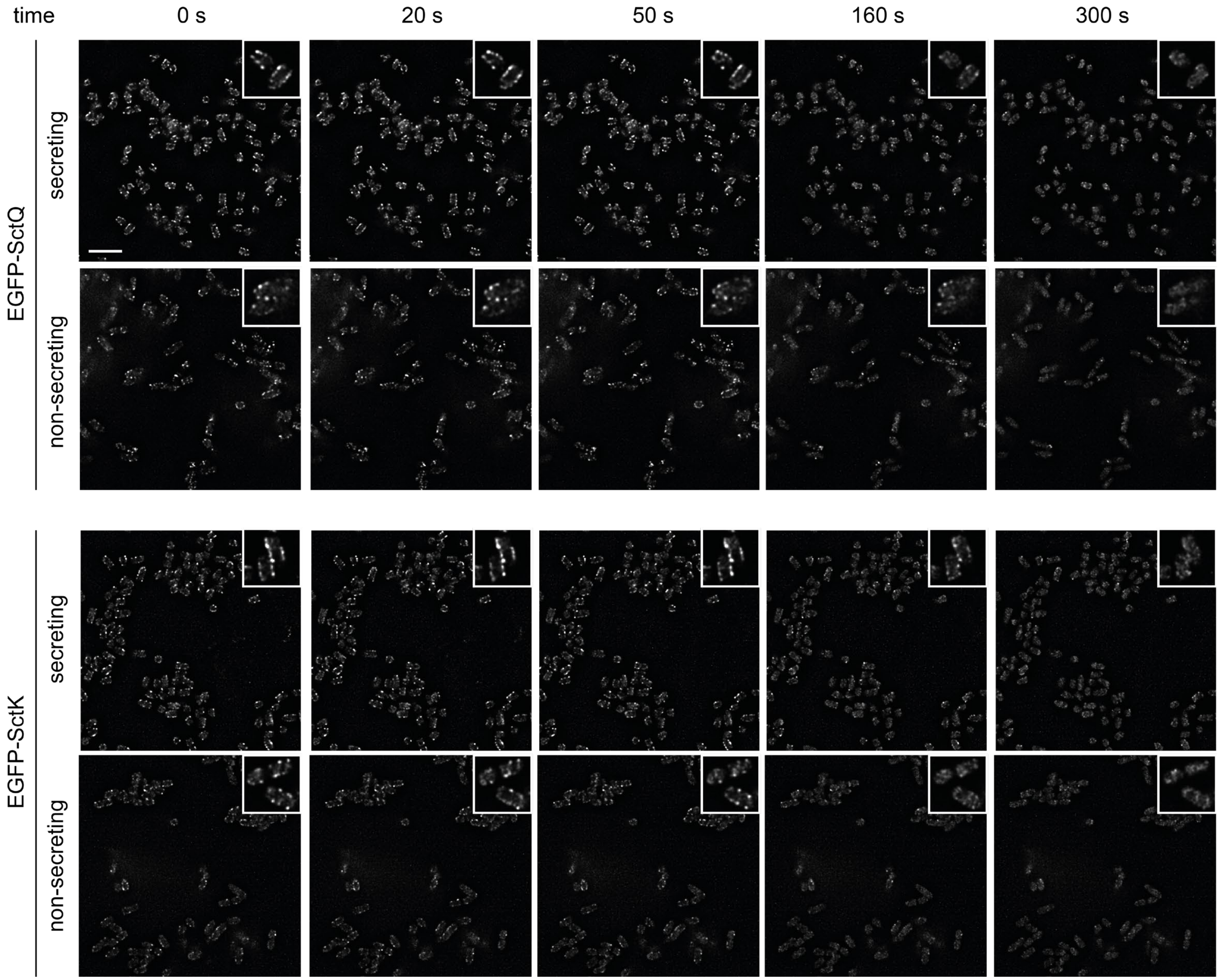

Supplementary Figure 8 – Dissociation kinetics of the cytosolic T3SS components under secreting and non-secreting conditions

Fluorescence micrographs of *Y. enterocolitica* EGFP-SctQ (top) or EGFP-SctK (bottom), at the given time periods after subjecting the bacteria to an external pH of 4 in a flow cell, under secreting conditions (rows 1 and 3), or non-secreting conditions (rows 2 and 4) Scale bar, 5 µm; insets show enlarged sections of the micrographs. *n*=3 independent experiments for EGFP-SctQ; *n*=2 independent experiment for EGFP-SctK.

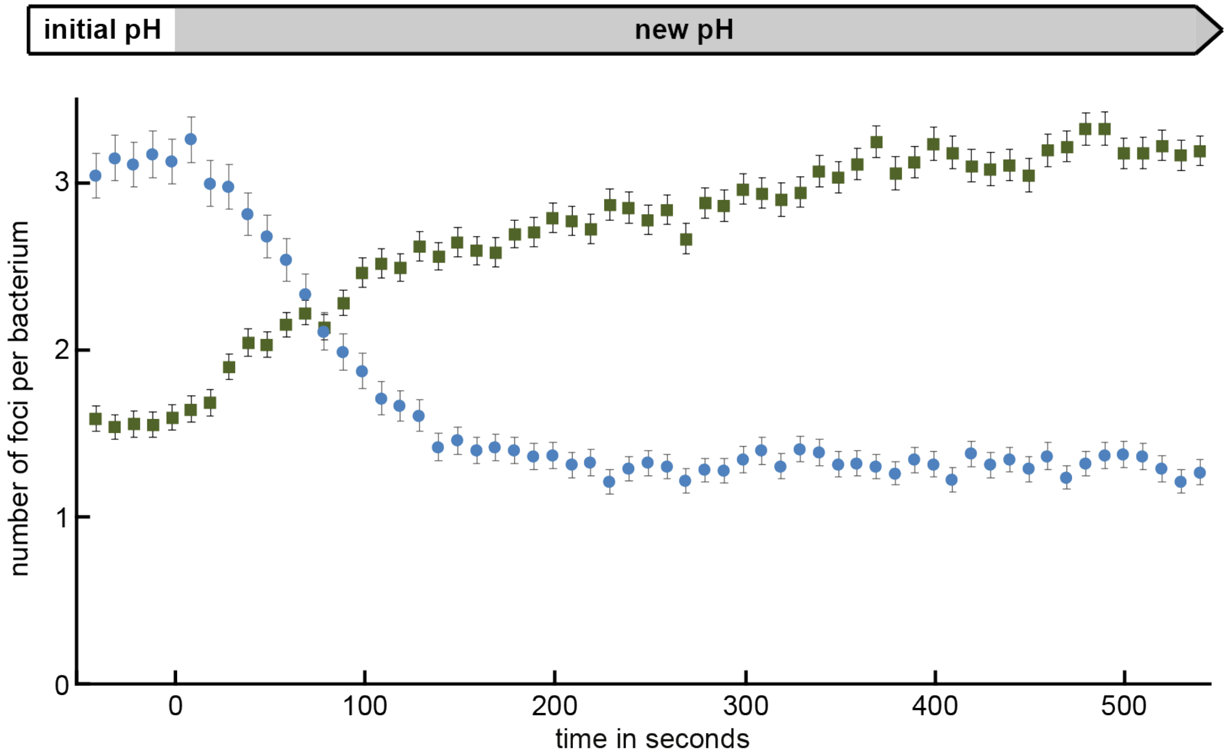

Supplementary Figure 9 – Quantification of EGFP-SctQ dissociation / re-association upon shift of external pH

The number of fluorescent EGFP-SctQ foci detected by BiofilmQ (see Material and Methods for details) was determined every 10 s in a flow cell upon changing the external pH at t=0, a) from 7 to 4 (blue circles; *n*=337-349 detected bacteria from 3 independent experiments), b) from 4 to 7 (green squares; *n*=328-347 detected bacteria from 3 independent experiments, except for t=40 s, where *n*=240 detected bacteria). Please refer to Source data file for number of bacteria at individual time points. Error bars display standard error of the mean. The exact distribution of foci per bacteria over time is listed in Supplementary Data 1.

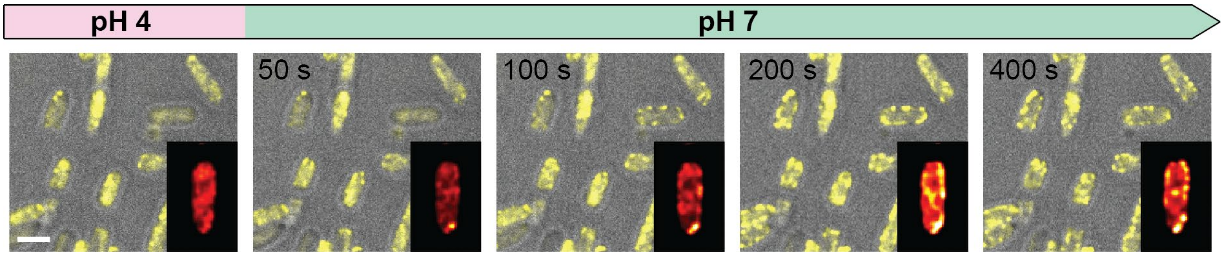

Supplementary Figure 10 – Re-association kinetics of EGFP-SctQ upon restoration of neutral external pH

Kinetics of EGFP-SctQ re-association after pH shift from 4 to 7. Overlay of phase contrast (grey) and fluorescence images (yellow); insets, enlarged single bacteria, visualized with the ImageJ red-hot color scale. *n*=3 independent experiments. Scale bar, 2 µm.

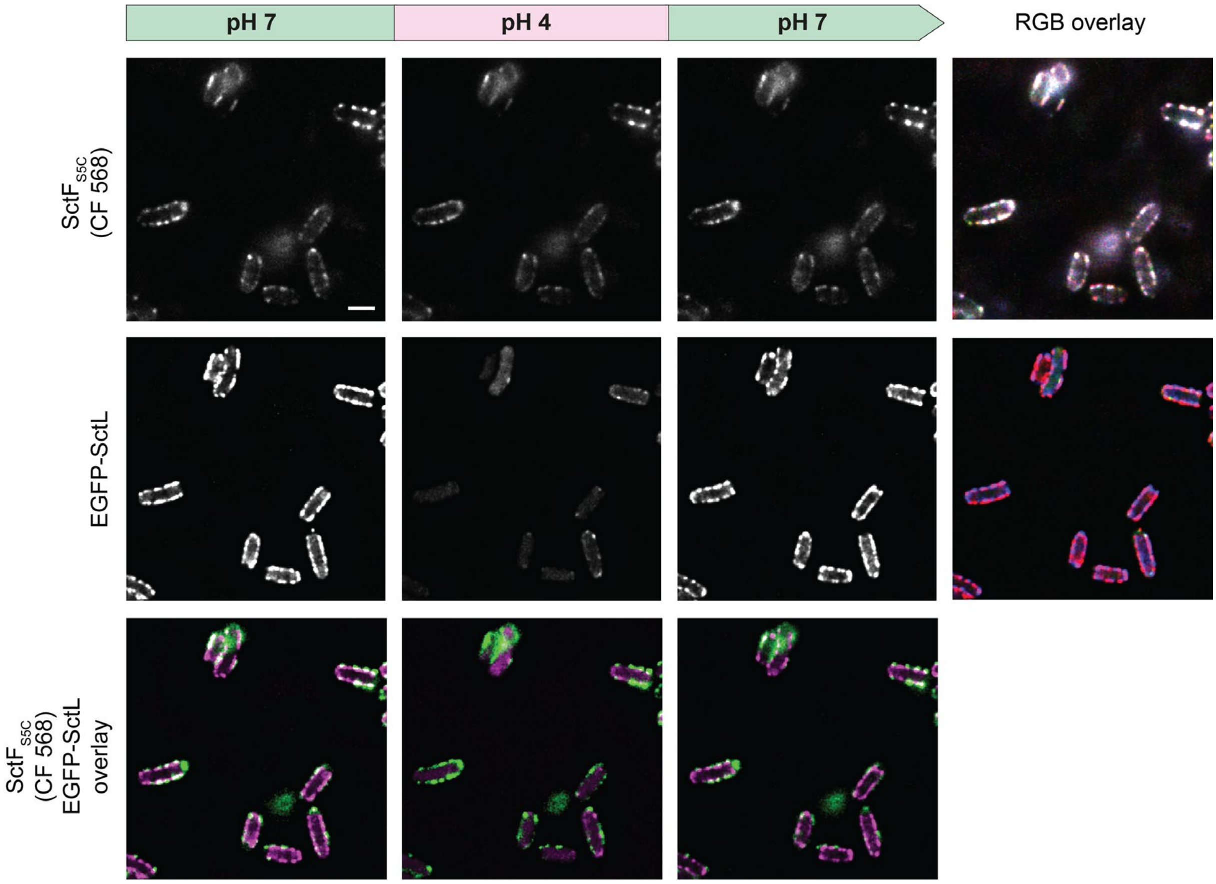

Supplementary Figure 11 – Localization of cytosolic T3SS subunits and the injectisome needle before and after incubation at pH 4

Fluorescence micrographs of *Y. enterocolitica* expressing EGFP-SctL (replacing the WT gene by allelic exchange) and $SctF_{S5C}$ (from plasmid) were subsequently taken at the indicated external pH values under secreting conditions. $SctF_{S5C}$ was labeled with maleimide-CF 568 and visualized in the red channel. Right column, composite images; red channel: pH 7 (first image on the left); green: pH 4 (second image on the left); blue: pH 7 (third image on the left). Bottom row, composite image of $SctF_{S5C}$ and mCherry-SctL (green and magenta, respectively). *n*=3 independent experiments. Scale bars, 2 µm.

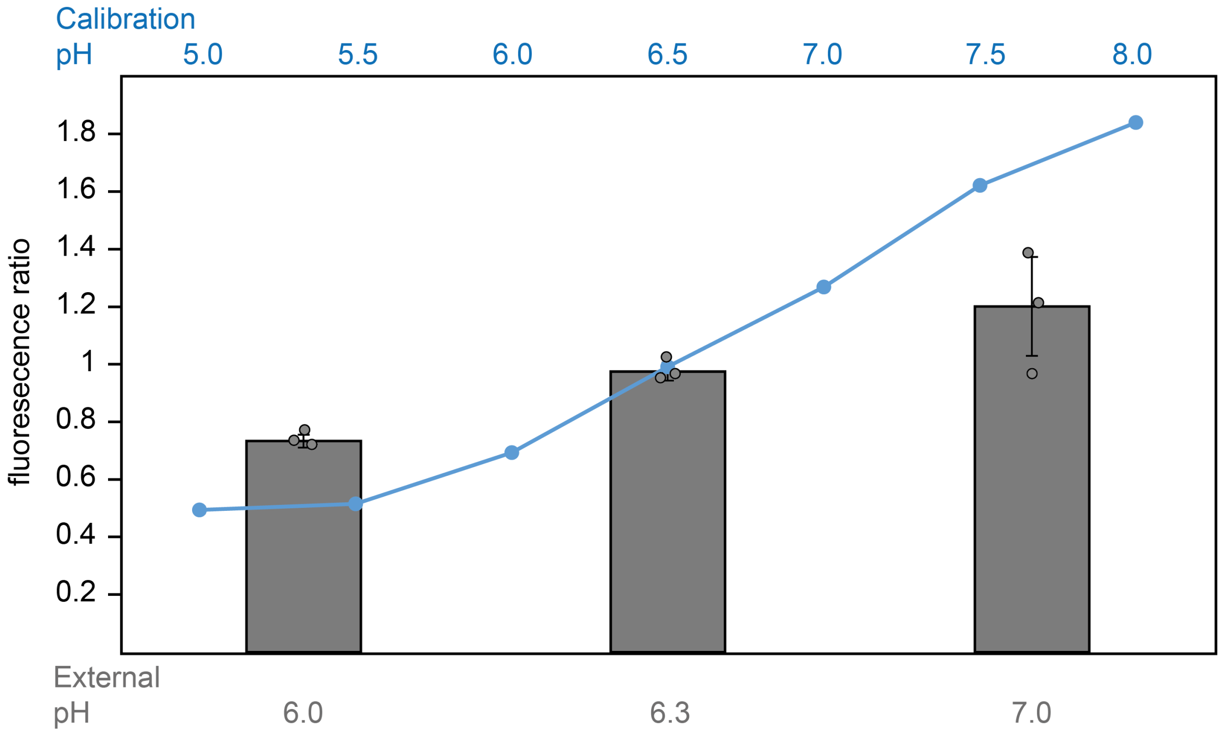

Supplementary Figure 12 – Internal pH is equilibrated with external pH upon DNP treatment

Blue curve, calibration of (Ex_{390nm} / Ex_{475nm}) fluorescence ratio of purified $pHluorin_{M153R}$ for the pH values indicated on the top (blue) (see Figure 3A). Technical triplicate, error bars too small to display. Grey bars, determination of cytosolic pH upon incubating bacteria at the indicated external pH values (bottom) in presence of 2 mM DNP. Fluorescence ratio (Ex_{390nm} / Ex_{475nm}) of bacteria expressing cytosolic $pHluorin_{M153R}$. *n*=3 fields of view from one representative experiment, single data points indicated by small black/grey circles. Bars denote mean values, error bars denote standard deviation.

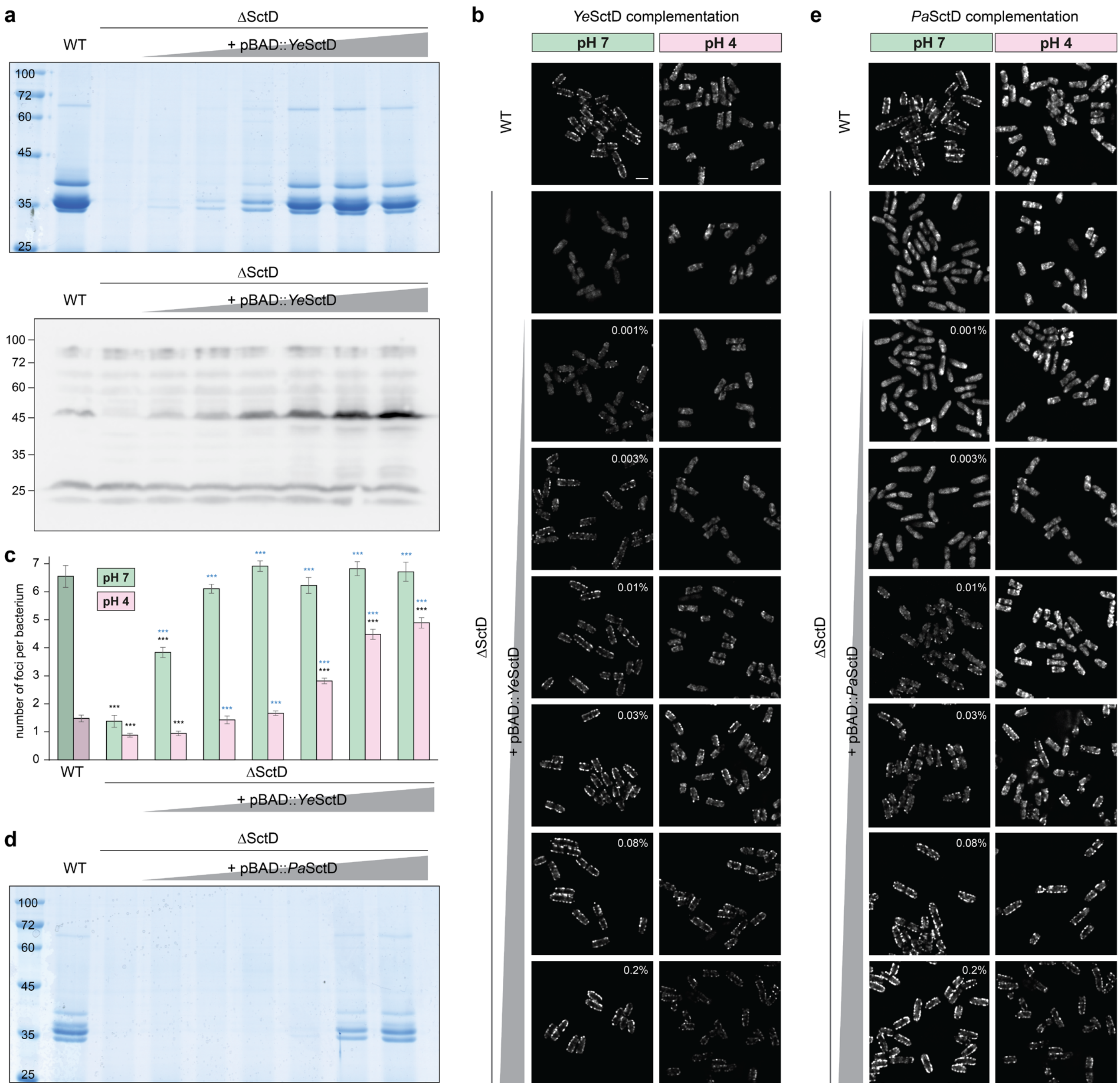

Supplementary Figure 13 – Influence of expression level of SctD on secretion and localization of the cytosolic components

(**a**) *In vitro* secretion assay (top) and immunoblot of total cellular protein anti-SctD (bottom) in EGFP-SctQ ΔSctD, complemented *in trans* by increasing induction levels of *Y. enterocolitica* SctD (from pBAD::*Ye*SctD, ramp indicates increasing arabinose concentrations as shown in (b)). Left side, molecular weight marker (kDa); expected molecular weight of *Ye*SctD, 46.9 kDa. (**b**) Localization of EGFP-SctQ in *Y. enterocolitica* EGFP-SctQ ΔSctD strains complemented *in trans* by *Ye*SctD. Percentages indicate arabinose concentration. Wild-type (WT) EGFP-SctQ shown as control at top. Scale bar, 2 µm. (**c**) Quantification of EGFP-SctQ foci per bacterium for the strains and conditions shown in (a) and (b). *n*=34/148, 63/251, 111/193, 153/108, 106/355, 53/361, 80/133, 49/101 bacteria (pH 7/pH 4 from top to bottom) from at three fields of view (four fields of view for 0.001% and 0.2% induction at pH 7, as well as 0.2% induction at pH 4) in a representative experiment. Bars denote mean values, error bars denote standard deviation; blue ***, $p<0.001$ against WT; black ***, $p<0.001$ against ΔSctD in a homoscedastic two-tailed t-test, other comparisons not statistically significantly different (see Source data file for individual p values). (**d**) *In vitro* effector secretion assay in EGFP-SctQ ΔSctD, complemented *in trans* by increasing induction levels of *P. aeruginosa* SctD (from pBAD::*Pa*SctD, ramp

indicates increasing arabinose concentrations as shown in (e)). (**e**) Localization of EGFP-SctQ in *Y. enterocolitica* EGFP-SctQ ΔSctD strains complemented *in trans* by *Pa*SctD. Percentages indicate arabinose concentration. Wild-type EGFP-SctQ shown as control at top. *n*=2 independent experiments comparing all strains (*n*>10 independent experiments for individual strains in different combinations). Scale bar, 2 µm.

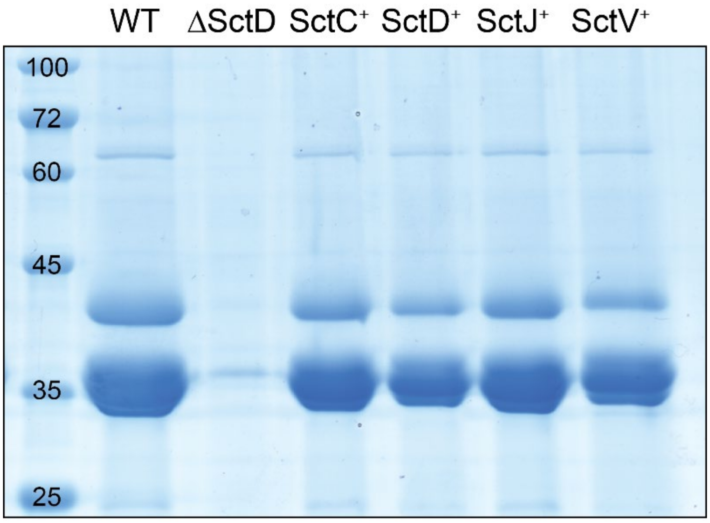

Supplementary Figure 14 – Secretion activity of the T3SS upon overexpression of membrane components

In vitro effector secretion assay in wild-type EGFP-SctQ strains and strains overexpressing the indicated proteins (induced by 0.2% arabinose), as shown in Figure 4bc. ΔSctD strain shown as a control. Left side, molecular weight marker (kDa). *n*=3 independent experiments.

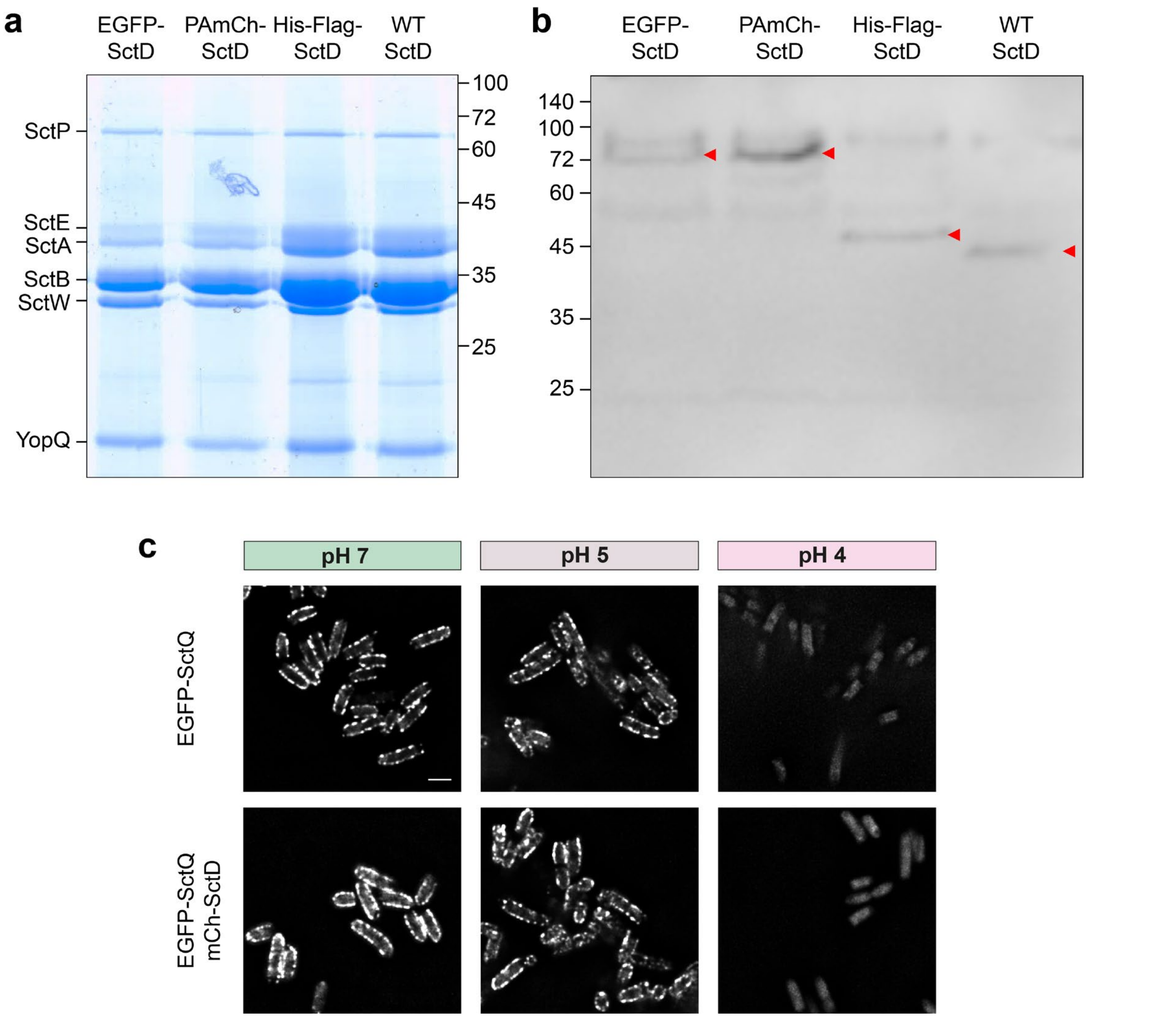

Supplementary Figure 15 – Functionality and stability of labeled SctD

(**a**) *In vitro* secretion assay showing the export of native T3SS substrates (indicated on left side) in the indicated strains. Supernatant of $3*10^8$ bacteria per lane; left side, assignment of exported proteins, right side, molecular weight marker (kDa). (**b**) Western blot using anti-SctD antibodies for total cellular protein of 2×10^8 bacteria per lane (strains as in (a)). Indicated proteins (expected molecular weight in kDa from left to right: 75.1, 74.9, 49.4, 46.7) indicated by red triangles. Left side, molecular weight marker (kDa). (**c**) Fluorescence micrographs of EGFP-SctQ in a strain otherwise wild-type (top) and in a strain also expressing mCherry-SctD (bottom) at the indicated external pH values. *n*=3 independent experiments. Scale bar, 2 µm.

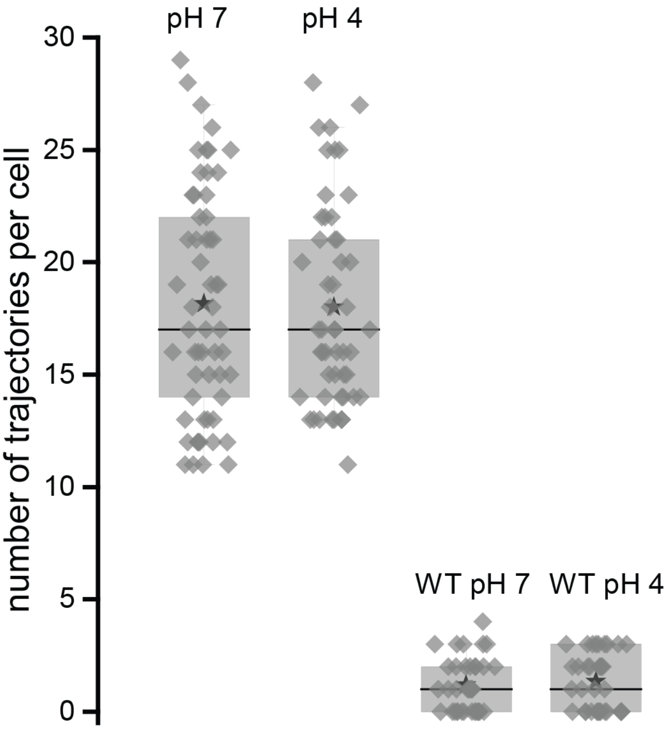

Supplementary Figure 16 – Number of SctD trajectories in *Y. enterocolitica* cells at pH 7 and pH 4 compared to the number of false positives measured in wild type cells

Number of PAmCherry-SctD trajectories per single living *Y. enterocolitica* cells at pH 7 and at pH 4. Both exhibit a medium trajectory number of 17 trajectories per cell and a mean of 18.2 $\pm$ 5.1 s.d. (pH 7) and 18.0 $\pm$ 4.4 s.d. (pH 4). As a control, strains expressing PAmCherry-SctD were mixed with wild type cells during the sample preparation. False positive trajectories from the background signal of single wild type cells in the same movies yield a median of one false positive trajectory per cell for both conditions and a mean of 1.2 $\pm$ 1.1 s.d. (pH 7) and 1.3 $\pm$ 1.2 s.d. (pH 4). Symbols in the histogram are black star mean, black line median, whisker range 5-95% and box range 25-75%.

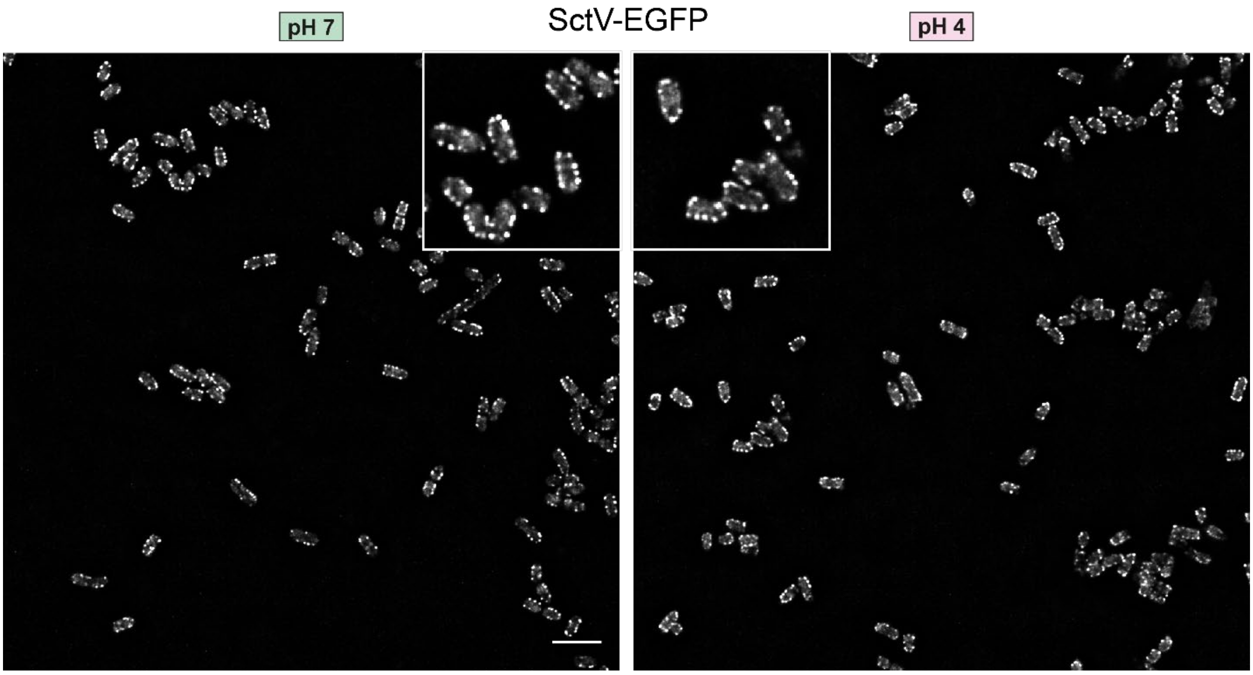

Supplementary Figure 17 - SctV-EGFP forms fluorescent foci at external pH of 7 and 4

Single micrographs of *Y. enterocolitica* SctV-EGFP at the indicated external pH. Scale bar, 5 µm. Insets 2x enlarged. *n*=3 independent experiments. See Figure 5c for RGB overlay of time course microscopy.

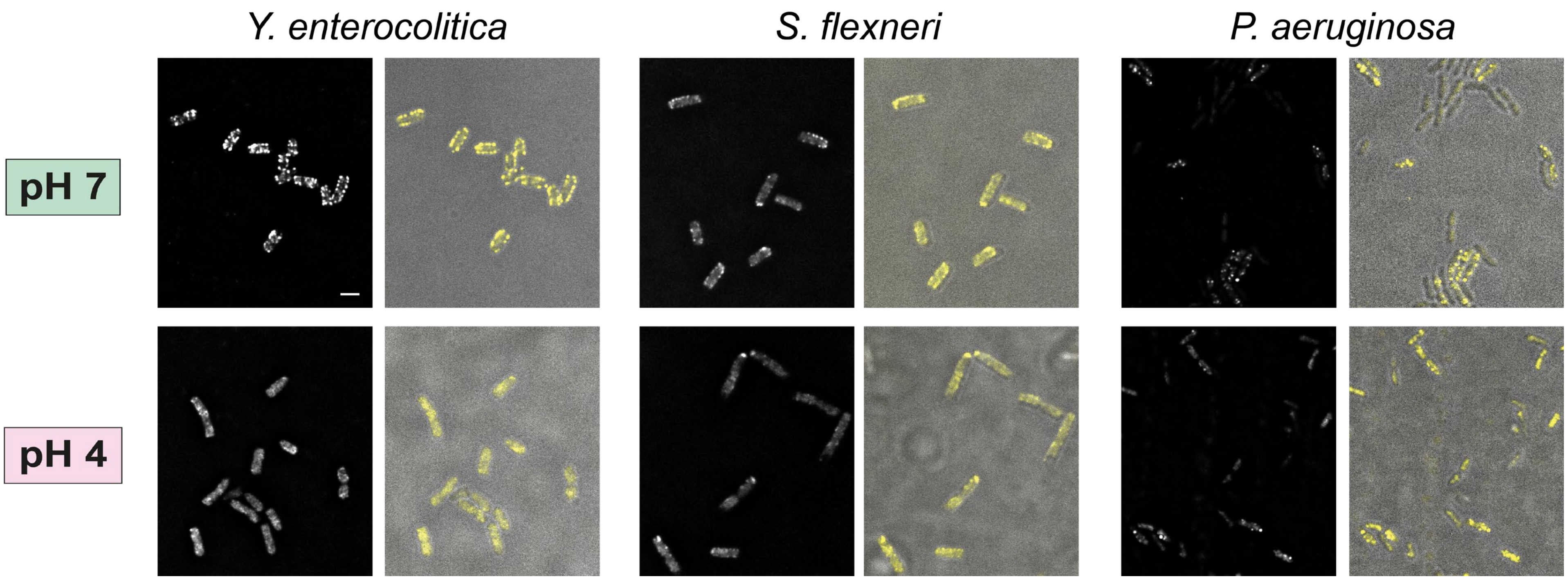

Supplementary Figure 18 – Low external pH decreases the fraction of bacteria with foci for fluorescently labeled cytosolic components in *Y. enterocolitica* and *S. flexneri*, but not in *P. aeruginosa*

Representative micrographs of *Yersinia enterocolitica* EGFP-SctQ, *Shigella flexneri* GFP-SctN, and *Pseudomonas aeruginosa* EGFP-SctQ at the indicated external pH values. Right side, overlays of phase contrast (grey) and fluorescence (yellow). Brightness of the *S. flexneri* images was increased two-fold to account for the lower stoichiometry of SctN in comparison to SctQ. *n*=3 independent experiments. Scale bar, 2 µm.

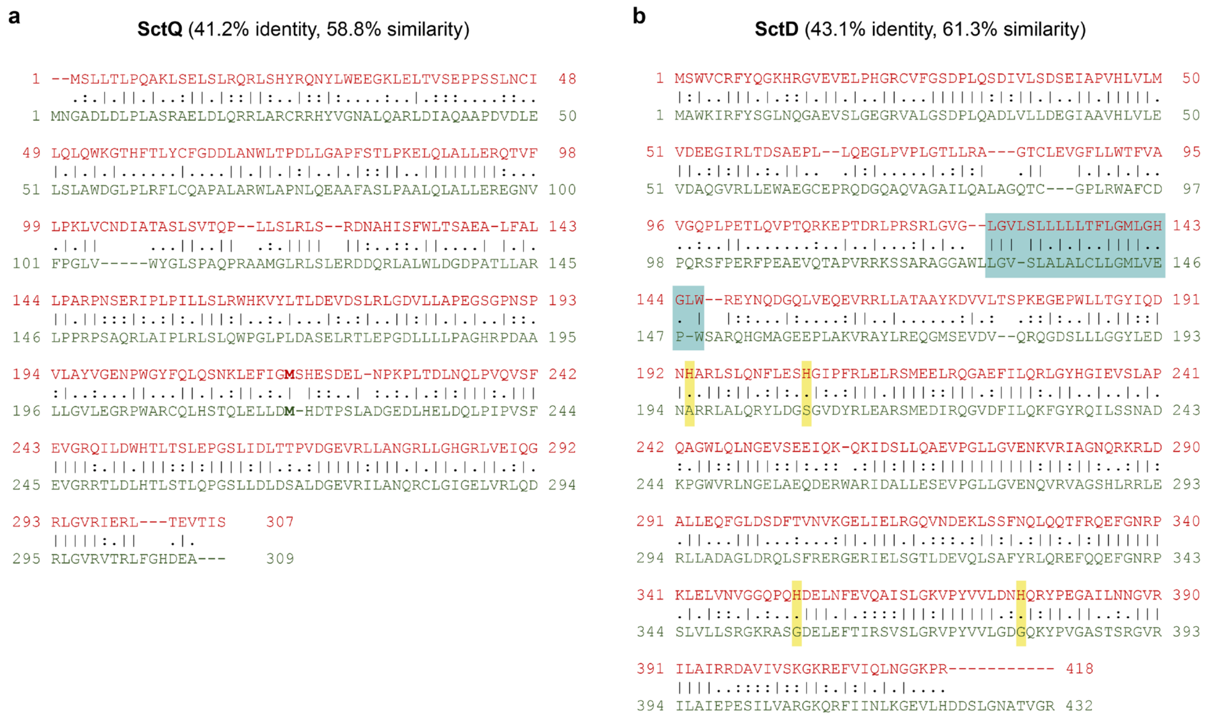

Supplementary Figure 19 – Sequence conservation of T3SS components in *Y. enterocolitica* and *P. aeruginosa*

Pairwise sequence alignment of SctQ (**a**) and SctD (**b**). Red, *Y. enterocolitica* sequences (NP_052404, NP_052414); green *P. aeruginosa* PAO1 sequences (NP_250385, NP_250408). Alignments created with EBI EMBOSS Needle (https://www.ebi.ac.uk/Tools/psa/emboss_needle/). Bold M indicates internal translation start site of $SctQ_C$ (amino acid 218 in *Y. enterocolitica*) [1,2]. Turquoise background indicates region of trans-membrane helix (TMH) for *Y. enterocolitica* SctD, as predicted by Phobius [3]. The regions upstream and downstream of the TMH correspond to the cytosolic and periplasmic parts of the structure, respectively. Amino acids highlighted in yellow were identified as potentially important for pH sensing in SctD. See Supplementary Table 1 for multi-species alignment including additional proteins used in this study.

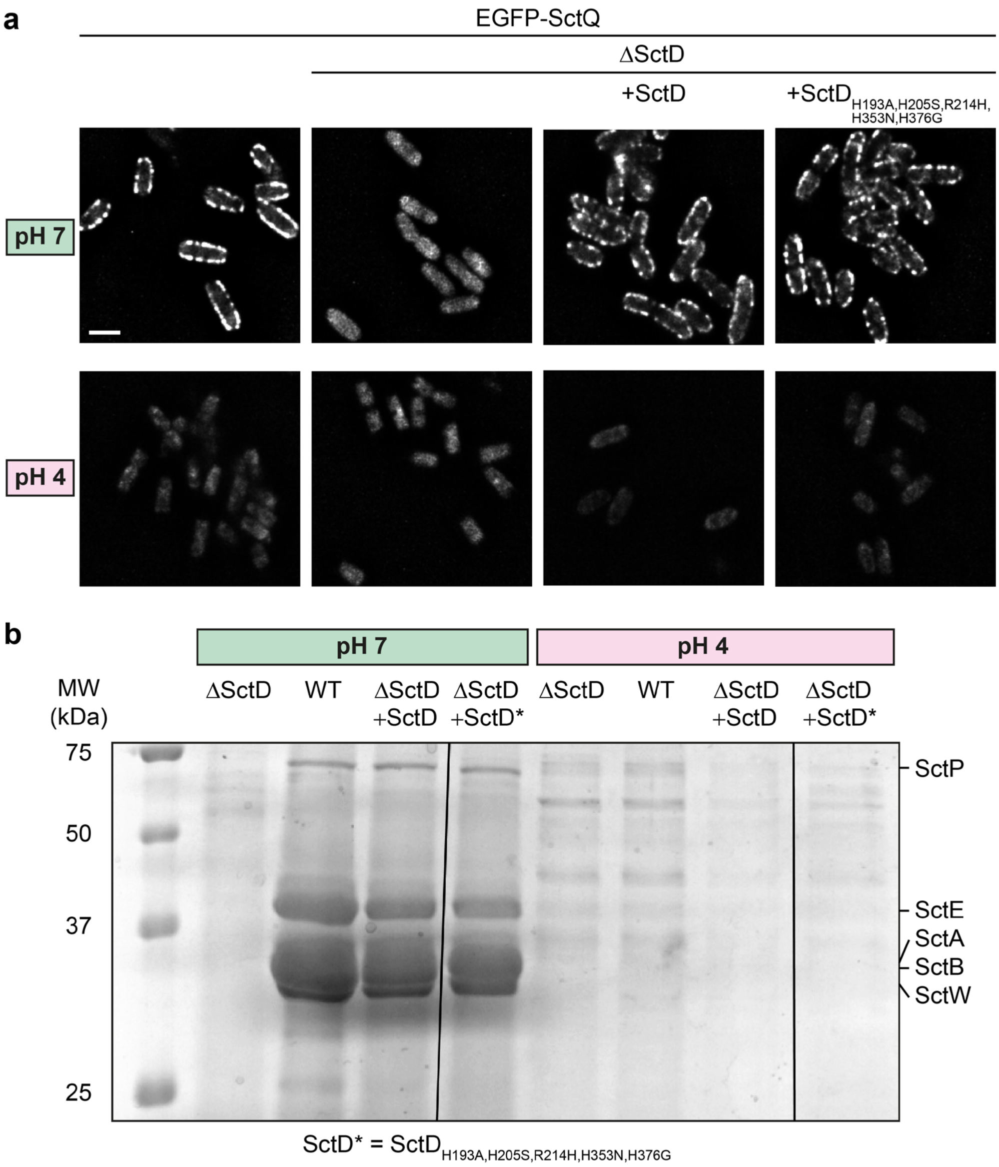

Supplementary Figure 20 – Point mutations in SctD do no suppress the pH-dependent dissociation of cytosolic T3SS components and suppression of secretion at low external pH

(**a**) Fluorescence micrographs of *Y. enterocolitica* EGFP-SctQ in strains lacking SctD (column 2-4) and complemented *in trans* with wild-type SctD (column 3) or an SctD multiple point mutant (see main text for details), at pH 7 (top) or pH 4 (bottom). The mutant SctD confers the same phenotype on SctQ localization as wild-type SctD under both conditions. Scale bar, 2 µm. (**b**) *In vitro* secretion assay showing the export of native T3SS substrates in the strains used in (a), at external pH of 7 (left) or 4 (right). All samples were analyzed on the same SDS-PAGE gel, vertical lines denote the omission of intermediate lanes. Molecular weight in kDa and exported proteins are indicated and the left and right side, respectively. Black and white scan of a coomassie-stained SDS-PAGE gel; supernatant of $3*10^8$ bacteria per lane. *n*=3 independent experiments.

Supplementary Tables

a

protein function	**Sct name**	*Yersinia pestis* EV NIIEG		*Pseudomonas aeruginosa* PAO-1		*Shigella flexneri* 2a str. 301		*Salmonella enterica subsp. enterica* serovar Typhimurium str. LT2; SPI-2			Protein length of *Y. enterocolitica* homolog
		**Identity (*similarity*) to *Y. enterocolitica* homolog in amino acids**									
secretin	**SctC**	594	*601*	370	*466*	158	*276*	155	*256*		607
outer MS ring protein	**SctD**	413	*418*	179	*261*	32	*60*	57	*100*		418
inner MS ring protein	**SctJ**	243	*243*	171	*204*	56	*98*	77	*129*		244
major export app. prot.	**SctV**	699	*702*	556	*630*	277	*441*	281	*435*		704
accessory cytos. prot.	**SctK**	206	*207*	84	*116*	9	*17*	*	*		209
C ring protein	**SctQ**	301	*305*	124	*178*	18	*31*	25	*36*		307
stator	**SctL**	208	*208*	115	*155*	n.d.	*n.d.*	35	*72*		223
ATPase	**SctN**	438	*439*	357	*385*	178	*256*	225	*275*		439

		**Identity (*similarity*) to *Y. enterocolitica homolog in %***								**Average identity**	***Average similarity***
secretin	**SctC**	97.9%	*99.0%*	61.0%	*76.8%*	26.0%	*45.5%*	25.5%	*42.2%*	52.6%	*65.9%*
outer MS ring protein	**SctD**	98.8%	*100.0%*	42.8%	*62.4%*	7.7%	*14.4%*	13.6%	*23.9%*	40.7%	*50.2%*
inner MS ring protein	**SctJ**	99.6%	*99.6%*	70.1%	*83.6%*	23.0%	*40.2%*	31.6%	*52.9%*	56.0%	*69.1%*
major export app. prot.	**SctV**	99.3%	*99.7%*	79.0%	*89.5%*	39.3%	*62.6%*	39.9%	*61.8%*	64.4%	*78.4%*
accessory cytos. prot.	**SctK**	98.6%	*99.0%*	40.2%	*55.5%*	4.3%	*8.1%*	*	*	35.8%	*40.7%*
C ring protein	**SctQ**	98.0%	*99.3%*	40.4%	*58.0%*	5.9%	*10.1%*	8.1%	*11.7%*	38.1%	*44.8%*
stator	**SctL**	93.3%	*93.3%*	51.6%	*69.5%*	n.d.	*n.d.*	15.7%	*32.3%*	40.1%	*48.8%*
ATPase	**SctN**	99.8%	*100.0%*	81.3%	*87.7%*	40.5%	*58.3%*	51.3%	*62.6%*	68.2%	*77.2%*

b

SctC

```
CLUSTAL O(1.2.4) multiple sequence alignment

Sf_SctC      --------MKKFNIKSLTLLIVLLPLIVNANNIDSHLLEQNDIAKYVAQSDTVGSFFERF      52
S2_SctC      -----MVVNKRLI---LIL--LFILNTAKSDELS----WKGNDFTLYARQMPLAEVLHLL      46
Ye_SctC      MAFPLHSFFKRVLTGTLLL----LSSYSWAQELD----WLPIPYVYVAKGESLRDLLTDF      52
Yp_SctC      MAFPLHSFFKRVLTGTLLL----LSNYSWAQELD----WLPIPYVYVAKGESLRDLLIDF      52
Pa_SctC      --------MRRLLIGGLLA--LLPGAVLRAQPLD----WPSLPYDYVAQGESLRDVLANF      46
                      ::.    *            :: :.             *:   : ..:  :

Sf_SctC      SALLNYPIVVSKQAAKKRISGEFDLSNPEEMLEKLTLLVGLIWYKDGNALYIYDSGELIS      112
S2_SctC      SENYDTAITISPL-ITATFSGKIPPGPPVDILNNLAAQYDLLTWFDGSMLYVYPASLLKH      105
Ye_SctC      GANYDATVVVSDK-INDKVSGQFEHDNPQDFLQHIASLYNLVWYYDGNVLYIFKNSEVAS      111
Yp_SctC      SANYDATVVVSDK-INDKVSGQFEHDNPQDFLQHIASLYNLVWYYDGNVLYIFKNSEVAS      111
Pa_SctC      GANYDASVIVSDK-VNDQVSGRFDLESPQAFLQLMASLYNLGWYYDGTVLYVFKTTEMQS      105
             .   :  : :*    .  .**.:    *  :*: ::   .*  : **. **::    :

Sf_SctC      KVILLENISLNYLIQYLKDANLYD-HRYPIRGNISDKTFYISGPPALVELVANTATLLDK      171
S2_SctC      QVITFNILSTGRFIHYLRSQNILSSPGCEVKEITGTKAVEVSGVPSCLTRISQLASVLDN      165
Ye_SctC      RLIRLQESEAAELKQALQRSGIWE-PRFGWRPDASNRLVYVSGPPRYLELVEQTAAALEQ      170
Yp_SctC      RLIRLQESEAAELKLALQRSGIWE-PRFGWRPDASNRLVYVSGPPRYLELVEQTAAALEQ      170
Pa_SctC      RLVRLEQVGEAELKRALTAAGIWE-ARFGWRADPSGRLVHVSGPGRYLELVEQTAQVLEQ      164
             ::: ::      :   *   .: .      :   . : . :**    :  : : *  *::

Sf_SctC      QVS--SIGTDKVNFGVIKLKNTFVSDRTYNMRGEDIVIPGVATVVERLLNNGKALSNRQA      229
S2_SctC      AL--IKRKDSAVSVSIYTLKYATAMDTQYQYRDQSVVVPGVVSVLREMSKTS-VPTSS-T      221
Ye_SctC      QTQIRSEKTGALAIEIFPLKYASASDRTIHYRDDEVAAPGVATILQRVLSDATIQQVT-V      229
Yp_SctC      QTQIRSEKTGALAIEIFPLKYASASDRTIHYRDDEVAAPGVATILQRVLSDATIQQVT-V      229
Pa_SctC      QYTLRSEKTGDLSVEIFPLRYAVAEDRKIEYRDDEIEAPGIASILSRVLSDANVVAVG-D      223
                  .   . : . :  *: : . *   . *.:.:  **:.::: .: . .

Sf_SctC      QNDPMPPFNITQKVSEDSNDFSFSSVTNSSILEDVSLIAYPETNSILVKGNDQQIQIIRD      289
S2_SctC      NNGS-----P-------------------ATQALPMFAADPRQNAVIVRDYAANMAGYRK      257
Ye_SctC      DNQRIPQAAT--------------------RASAQARVEADPSLNAIIVRDSPERMPMYQR      270
Yp_SctC      DNQRIPQAAT--------------------RASAQAKVEADPSLNAIIVRDSPERMPMYQR      270
Pa_SctC      EPGKLRP--G--------------------PQSSHAVVQAEPSLNAVVVRDHKDRLPMYRR      262
             :                                    . * *  *:::*:.   .:   :

Sf_SctC      IITQLDIAKRHIELSLWIIDIDKSELNNLGVNWQGTASFGDSFGASFNM-----------      338
S2_SctC      LITELDQRQQMIEISVKIIDVNAGDINQLGIDWGTAVSLGGKKIAFNTG----LNDG---      310
Ye_SctC      LIHALDKPSARIEVALSIVDINADQLTELGVDWRVGIRTGNNHQVVIKTTGDQSN---IA      327
Yp_SctC      LIHALDKPSARIEVALSIVDINADQLTELGVDWRVGIRTGNNHQVVIKTTGDQSN---IA      327
Pa_SctC      LIEALDRPSARIEVGLSIIDINAENLAQLGVDWSAGIRLGNNKSIQIRTTGQDSEEGGGA      322
             :*  **  .  **:.: *:*::  :: :**::*      *..

Sf_SctC      ---SSSASISTLDGNKFIASVMALNQKKKANVVSRPVILTQENIPAIFDNNRTFYVSLVG      395
S2_SctC      --GASGFSTVISDTSNFMVRLNALEKSSQAYVLSQPSVVTLNNIQAVLDKNITFYTKLQG      368
Ye_SctC      SNGALGSLVDARGLDYLLARVNLLENEGSAQVVSRPTLLTQENAQAVIDHSETYYVKVTG      387
Yp_SctC      SNGALGSLIDARGLDYLLARVNLLENEGSAQVVSRPTLLTQENAQAVIDHHETYYVKVTG      387
Pa_SctC      GNGAVGSLVDSRGLDFLLAKVTLLQSQGQAQIGSRPTLLTQENTQAVLDQSETYYVRVTG      382
                :  .      . .  ::.  :  *:..  .* :  *:*  ::* :*  *::*:  *:*. : *

Sf_SctC      ERNSSLEHVTYGTLINVIPRFSSRG---QIEMSLTIEDGTGNSQSNYNYNNENTSVLPEV      452
S2_SctC      EKVAKLESITTGSLLRVTPRLLNDNGTQKIMLNLNIQDGQQSD------TQSETDPLPEV      422
Ye_SctC      KEVAELKGITYGTMLRMTPRVLTQGDKSEISLNLHIEDGNQKP------NSSGIEGIPTI      441
Yp_SctC      KEVAELKGITYGTMLRMTPRVLTQGDKSEISLNLHIEDGNQKP------NSSGIDGIPTI      441
Pa_SctC      ERVAELKAITYGTMLKMTPRVVTLGDTPEISLSLHIEDGSQKP------NSAGLDKIPTI      436
             :. :.*: :* *:::.: **. . .   :* :.* *:**  .       ..   . :* :

Sf_SctC      GRTKISTIARVPQGKSLLIGGYTHETNSNEIISIPFLSSIPVIGNVFKYKTSNISNIVRV      512
S2_SctC      QNSEIASQATLLAGQSLLLGGFKQGKQIHSQNKIPLLGDIPVVGHLFRNDTTQVHSVIRL      482
Ye_SctC      SRTVVDTVARVGHGQSLIIGGIYRDELSVALSKVPLLGDIPYIGALFRRKSELTRRTVRL      501
Yp_SctC      SRTVVDTVARVGHGQSLIIGGIYRDELSVALSKVPLLGDIPYLGALFRRKSELTRRTVRL      501
Pa_SctC      NRTVIDTIARVGHGQSLLIGGIYRDELSQSQRKVPWLGDIPYLGALFRTTADTVRRSVRL      496
              .: : : * :  *:**::**  :        .:* *..** :* :*:  :      :*:
```

```
Sf_SctC      FLIQPREIKESSYYNTAEYKSLISEREIQKTT----QIIPSETT---L--------LEDE      557
S2_SctC      FLIKASVVNNGISHG---------------------------------------------      497
Ye_SctC      FIIEPRIIDEGIAHHLA----LGNGQDLRTGILTVDEISNQSTTLNKLLGGSQCQPLNKA      557
Yp_SctC      FIIEPRIIDEGIAHHLA----LGNGRDLRTGILAVDEISNQSTTLNKLLGGFQCQPLNKA      557
Pa_SctC      FLIEPRLIDDGVGHYLA----LNNRRDLRGGLLEIDELSNQSLSLRKLLGSARCQALAPA      552
             *:*:   :.:.  :

Sf_SctC      KSL---------VSYLNY--------------------------------   566
S2_SctC      --------------------------------------------------   497
Ye_SctC      QEVQKWLSQNNKSSYLTQCKMDKSLGWRVVEGACTPAQSWCVSAPKRGVL   607
Yp_SctC      QEVQKWLSQNNKSSYLTQCKMDKSLGWRVVEGACTPAESWCVSAPKRGVL   607
Pa_SctC      RAEQERLRQAGQGSFLTPCRMGAQEGWRVTDGACPKDGAWCVGAERGN--   600
```

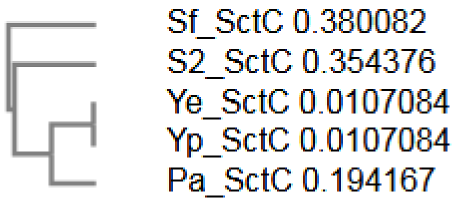

SctD

```
CLUSTAL O(1.2.4) multiple sequence alignment

Sf_SctD      ----MSEAKNSNLAPFRLLVKLTNGVGDEFPLYYGNNLIVLGRTIETLEFGNDNFPENII      56
S2_SctD      MAYLMVNPKSSWK--IRF----LGHVLQGREVWLNEG---------NLSLGEKGCDICIP      45
Pa_SctD      ---------MAWK--IRF----YSGLNQGAEVSLGEG---------RVALGSDPLQADLV      36
Ye_SctD      ---------MSWV--CRF----YQGKHRGVEVELPHG---------RCVFGSDPLQSDIV      36
Yp_SctD      ---------MSWV--CRF----YQGKHRGVEVELPHG---------RCVFGSDPLQSDIV      36
                       :     *:             :   ..            :*..     :

Sf_SctD      PVTDSKSDGIIYLTISKDNICQFSDEKGEQIDIN--------------------------      90
S2_SctD      L-AINE---KIIL--REQADSLFVDAGKARVRVNGRRFNP---NKPLPSSGVLQVAGVAI      96
Pa_SctD      L-LDEG---IAA-----VHLVLEVDAQGVRLLEWAEGCEPRQDGQAQVAGAILQ-ALAGQ      86
Ye_SctD      L-SDSE---IAP-----VHLVLMVDEEGIRLTDSAEPL--LQEGLPVPLGTLLR-AGTCL      84
Yp_SctD      L-SDSE---IAP-----VHLVLMVDEEGIRLTDSAEPL--LQEGLPVPLGTLLR-AGSCL      84
                 .                   *    ::

Sf_SctD      ---SQFNSFEYDGISFH----------LKNMREDKSRGHILNGMYKNHSVFFFFAV--IV      135
S2_SctD      AFGKQD--------------------CELADYQIPVSRS----GYWWLAGVFLIFIGG-MG      132
Pa_SctD      TCGPLRWAFCDPQRSFPERFPEAEVQTAPVRRKSSARA----GGAWLLGVSLALALCLLG      142
Ye_SctD      EVGFLLWTFVAVGQPLPETLQVPTQRKEPTDRLPRSRL----GVG-LGVLSLLLLLTFLG      139
Yp_SctD      EVGFLLWTFVAVGQPLPETLQVPTQRKEPTDRLPRSRL----GIG-LGVLSLLLLLTFLG      139
                                            :   :*     *      : : :    :

Sf_SctD      VLII-IFSLSLKKD-----EVKEIAEIIDDKRYGIVNTGQCNYILAETQNDAVWASVA--      187
S2_SctD      VLLS----ISGQPETVNDLPLRV-KFLLDK--------SNIHYVRAQWKEDGSLQLSGYC      179
Pa_SctD      MLVE-PWSARQ-HGMAGEEPLAKVRAYLRE--------QGMS-EVDVQRQGDSLLLGGYL      191
Ye_SctD      MLGHGLWREYNQDGQLVEQEVRRLLATAAY--------KDVV-LTSPK-EGEPWLLTGYI      189
Yp_SctD      LLGHGLWREYNQDGQLVEQEVRRLLATAAY--------KDVV-LTSPKKEGEPWLLTGYI      190
             :*                  :                            :.      .

Sf_SctD      ------------LNKTGFTKCRYILVSNKEINRIQQYINQRFPFINLYVLNLVSDKAELL      235
S2_SctD      SSSEQMQKVRATLESWG-VMYRDGVICDDLLVREVQDVLIKMGYPHAEVSSE---GPGS-      234
Pa_SctD      EDNARRLALQRYLDGSG-VDYRLEARSMEDIRQGVDFILQKFGYRQILSSNAD--KPGW-      247
Ye_SctD      QDNHARLSLQNFLESHG-IPFRLELRSMEELRQGAEFILQRLGYHGIEVSLAP--QAGW-      245
Yp_SctD      QDNHARLSLQNFLESHG-IPFRLELRSMEELRQGAEFILQRLGYHGIEVSLAP--QAGW-      246
                         *:  *    *    . . : :  : :  :: :

Sf_SctD      VFLSKERNSSKDTELDKLKNALIVEFPYIKNIKFNY--LSDHNARGDA----KGIFTKVN      289
S2_SctD      VLIHD-DIQ-MDQQWRKVQPLL-ADIPGLLHWQISHSHQSQGDDIISA-IIENGLVGLVN      290
Pa_SctD      VRLNG-ELAEQDERWARIDALLESEVPGLLGVENQVRVAGSHLRRLERLLADAGLDRQLS      306
Ye_SctD      LQLNG-EVSEEIQ-KQKIDSLLQAEVPGLLGVENKVRIAGNQRKRLDALLEQFGLDSDFT      303
Yp_SctD      LQLNG-EVSEEIQ-KQKIDSLLQAEVPGLLGVESKVRIAGNQRKRLDALLEQFGLDSDFT      304
             : :             ::.  *  :.* :   : .    ..     .      *:    ..

Sf_SctD      VQYKEICENNKVTYSVREELTDEKLELINRLISEHKNIYGDQYIEFSVLLID-----D--      342
S2_SctD      VTP------MRRSFVISGVLDESHQ---RILQETLAALKKKDPA-LSLIYQDIAPSHDES      340
Pa_SctD      FRE------RGERIELSGTLDEVQLSAFYRLQREFQQEFGNRPS-LVLLSRGKRASGDEL      359
Ye_SctD      VNV------KGELIELRGQVNDEKLSSFNQLQQTFRQEFGNRPK-LELVNVGGQPQHDEL      356
Yp_SctD      VNV------KGELIELRGQVNDEKLNSFNQLQQTFRQEFGNRPK-LELVNVGGQPQHDEL      357
             .              :   : : :      *         .    : ::  .     *

Sf_SctD      DFKGKSY----LNSKDSYVMLNDKHWFFLDKNK---------------------------      371
S2_SctD      KYLPAPVAGFVQSRHGNYLLLTNKERLRVGALLPNGGEIVHLSADVVTIKHYDTLINYPL      400
Pa_SctD      EFTIRSV----SLGRVPYVVLGDGQKYPVGASTSRGVRILAIEPESILVARGKQR--FII      413
Ye_SctD      NFEVQAI----SLGKVPYVVLDNHQRYPEGAILNNGVRILAIRRDAVIVSKGKRE--FVI      410
Yp_SctD      NFEVQAI----SLGKVPYVVLDNHQRYPEGAILNNGVRILAIRRDAVIVSKGKRE--FVI      411
             .:            :  *::* : .    .

Sf_SctD      ------------------- 371
S2_SctD      DFK---------------- 403
Pa_SctD      NLKGEVLHDDSLGNATVGR 432
Ye_SctD      QLNGGKPR----------- 418
Yp_SctD      QLNGGKPR----------- 419
```

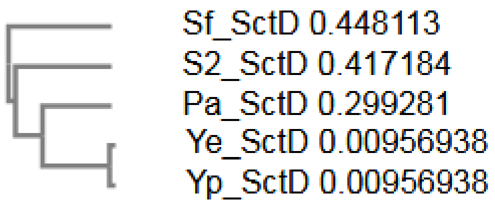

SctJ

```
CLUSTAL O(1.2.4) multiple sequence alignment

Sf_SctJ         -----MIRYKGFILFLLLMLIGCEQREELISNLSQRQANEIISVLERHNITARKVDGGKQ      55
S2_SctJ         ----MKVHRIVFLTVLTFFLTACD--VDLYRSLPEDEANQMLALLMQHHIDAEKKQEEDG      54
Pa_SctJ         MRRTVKGLSRMALLALVLALGGCK--VELYTGISQKEGNEMLALLRSEGVSADKQADKDG      58
Ye_SctJ         ----MKVKTSLSTLILILFLTGCK--VDLYTGISQKEGNEMLALLRQEGLSADKEPDKDG      54
Yp_SctJ         ----MKVKTSLSTLILILFLTGCK--VDLYTGISQKEGNEMLALLRQEGLSADKEPDKDG      54
                               * : * .*.   :*  .: : :.*:::::*  . : * *    .

Sf_SctJ         GISVQVEKGTFASAVDLMRMYDLPNPERVDISQMFPTDSLVSSPRAEKARLYSAIEQRLE      115
S2_SctJ         -VTLRVEQSQFINAVELLRLNGYPHRQFTTADKMFPANQLVVSPQEEQQKINFLKEQRIE      113
Pa_SctJ         TVRLLVEESDIAEAVEVLKRKGYPRENFSTLKDVFPKDGLISSPIEERARLNYAKAQEIS      118
Ye_SctJ         KIKLLVEESDVAQAIDILKRKGYPHESFSTLQDVFPKDGLISSPIEELARLNYAKAQEIS      114
Yp_SctJ         KIKLLVEESDVAQAIDILKRKGYPHESFSTLQDVFPKDGLISSPIEELARLNYAKAQEIS      114
                 : : **:. . .*:::::  . *. .    ..:** : *: **  *  ::     *.:.

Sf_SctJ         QSLVSIGGVISAKIHVSYDLEEKNISS--KPMHISVIAIYDSPKESELLVSNIKRFLKNT      173
S2_SctJ         GMLSQMEGVINAKVTIALPTYDE--GSNASPSSVAVFIKYSPQVNMEAFRVKIKDLIEMS      171
Pa_SctJ         HTLSEIDGVLVARVHVVLPEERDGLGRKSSPASASVFIKHAADVQLDAYVPQIKQLVNNG      178
Ye_SctJ         RTLSEIDGVLVARVHVVLPEEQNNKGKKGVAASASVFIKHAADIQFDTYIPQIKQLVNNS      174
Yp_SctJ         RTLSEIDGVLVARVHVVLPEEQNNKGKKGVAASASVFIKHAADIQFDTYIPQIKQLVNNS      174
                  * .: **: *:: :      .  .        :*:  :    : :    :** :::

Sf_SctJ         FSDVKYENISVILTPKEEYVYTNVQPVKEV----------KSEFLTNEVIYLFLGMAVLV      223
S2_SctJ         IPGLQYSKISILMQPA-EFRMVADVPARQTFWIMDVINANKGKVVKWLMKYPYPLMLSLT      230
Pa_SctJ         IEGLSYDRISVVLVPSAGVRQVPLAPRFESVFSIQVAEHSRGRLLGLF---------GLL      229
Ye_SctJ         IEGLAYDRISVILVPSVDVRQSSHLPRNTSILSIQVSEESKGRLIGLL---------SLL      225
Yp_SctJ         IEGLAYDRISVILVPSVDVRQSSHLPRNTSILSIQVSEESKGHLIGLL---------SLL      225
                : .: *..**::: *         *              :...:              *

Sf_SctJ         VILLVWAFKTG--WFKRNKI      241
S2_SctJ         GLLLGVGILIGYFCLRRRF-      249
Pa_SctJ         LALLLASNLAQFFWHRQRG-      248
Ye_SctJ         ILLLPVTNLAQYFWLQRKK-      244
Yp_SctJ         ILLLPVTNLAQYFWLQRKK-      244
                  **          ::.
```

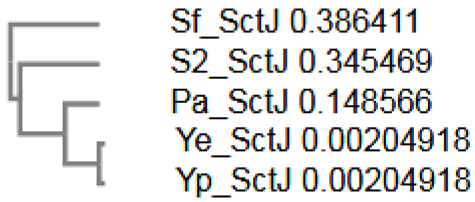

SctV

```
CLUSTAL O(1.2.4) multiple sequence alignment

Sf_SctV         ---------MIQSFLKQVSTKPELIIILVLMVMIIAMLIIPLPTYLVDFLIGLNIVLAILV      51
S2_SctV         MRSWLGEGVRAQQWLSVCAGRQDMVLATVLLIAIVMMLLPLPTWMVDILITINLMFSVIL      60
Pa_SctV         -------MNDLSGLLGRVGERKDILLVVLLLAVVFMMVLPLPPLLLDILIAVNITISVVL      53
Ye_SctV         ------MNPHDLEWLNRIGERKDIMLAVLLLAVVFMMVLPLPPLVLDILIAVNMTISVVL      54
Yp_SctV         ------MNPHDLEWLNRIGERKDIMLAVLLLAVVFMMVLPLPPLVLDILIAVNMTISVVL      54
                              *   . : :::: .:::  : *:::***  ::*:** :*: :::::

Sf_SctV         FMGSFYIERILSFSTFPSVLLITTLFRLALSISTSRLILVDADAGKIITTFGQFVIGDSL      111
S2_SctV         LLIAIYLSDPLDLSVFPSLLLITTLYRLSLTISTSRLVLLQHNAGNIVDAFGKFVVGGNL      120
Pa_SctV         LMMSVYIGSPLQFSVFPAVLLITTLFRLALSVSTTRMILLQADAGQIVNTFGSFVVGGNL      113
Ye_SctV         LMIAIYINSPLQFSAFPAVLLVTTLFRLALSVSTTRMILLQADAGQIVYTFGNFVVGGNL      114
Yp_SctV         LMIAIYINSPLQFSAFPAVLLVTTLFRLALSVSTTRMILLQADAGQIVYTFGNFVVGGNL      114
                :: :.*:   *.:*.**::**:***:**:*::**:*::*:: :**:*: :**.**:*..*

Sf_SctV         AVGFVIFSIVTVVQFIVITKGSERVAEVAARFSLDGMPGKQMSIDADLKAGIIDAAGAKE      171
S2_SctV         TVGLVVFTIITIVQFIVITKGIERVAEVSARFSLDGMPGKQMSIDGDLRAGVIDADHART      180
Pa_SctV         VVGIIIFLIITIVQFLVITKGAERVAEVSARFSLDAMPGKQMSIDGDMRAGVIDVNEARA      173
Ye_SctV         IVGIVIFLIITIVQFLVITKGSERVAEVSARFSLDAMPGKQMSIDGDMRAGVIDVNEARE      174
Yp_SctV         IVGIVIFLIITIVQFLVITKGSERVAEVSARFSLDAMPGKQMSIDGDMRAGVIDVNEARE      174
                 **:::* *:*:***:***** *****:******.*********.*::**:**.   *:

Sf_SctV         RRSILERESQLYGSFDGAMKFIKGDAIAGIIIIFVNLIGGISVGMSQHGMSLSGALSTYT      231
S2_SctV         LRQHVQQESRFLGAMDGAMKFVKGDTIAGIIVVLVNIIGGIIIAIVQYDMSMSEAVHTYS      240
Pa_SctV         RRAVIEKESQMFGSMDGAMKFVKGDAIAGLIIIVVNILGGIAIGVTQKGLSTADALQLYA      233
Ye_SctV         RRATIEKESQMFGSMDGAMKFVKGDAIAGLIIIFVNILGGVTIGVTQKGLAAAEALQLYS      234
Yp_SctV         RRATIEKESQMFGSMDGAMKFVKGDAIAGLIIIFVNILGGVTIGVTQKGLAAAEALQLYS      234
                 *  :::**:: *::******:***:***:*::.**::**: :.: * .:: : *:  *:

Sf_SctV         ILTIGDGLVSQIPALLISISAGFIVTRVNGD-SDNMGRNIMSQIFGNPFVLIVTSALALA      290
S2_SctV         VLSIGDGLCGQIPSLLISLSAGIIVTRVPGEKRQNLATELSSQIARQPQSLILTAVVLML      300
Pa_SctV         VLTVGDGMVSQVPALLIAITAGIIVTRVSSDESADLGSDIGEQVVAQPKALLIGGLLLVL      293
Ye_SctV         ILTVGDGMVSQVPALLIAITAGIIVTRVSSEDSSDLGSDIGKQVVAQPKAMLIGGVLLLL      294
Yp_SctV         ILTVGDGMVSQVPALLIAITAGIIVTRVSSEDSSDLGSDIGKQVVAQPKAMLIGGVLLLL      294
                :*::***: .*:*:***:::**:***** .:   ::. :: .*:  :*  ::: . : :

Sf_SctV         IGMLPGFPFFVFFLIAVTLTALFYYKKVVEKEKSL------SESDSSGYTGTFDI-----      339
S2_SctV         LALIPGFPFITLAFFSALLALPIILIRRK-KSVVS----------ANGVEAPEK--DSMV      347
Pa_SctV         FGLIPGFPTLTFLALALLVGGGGYFMLWRQRAQASAGSRDLPALLAQGAGAPSAKARGKA      353
Ye_SctV         FGLIPGFPTVTFLILALLVGCGGYMLSRKQ-SRNDEANQDLQSILTSGSGAPAARTKAKT      353
Yp_SctV         FGLIPGFPTVTFLILALLVGCGGYMLSRKQ-SRNDEANQDLQSILTSGSGAPAARTKAKT      353
                :.::**** ..:  ::  :                          :.*  .

Sf_SctV         --DNSHDSSLAMIENLDAISSETVPLILLFAE---NKINANDMEGLIERIRSQFFIDYGV      394
S2_SctV         PG--------------------ACPLILRLSP----TLHSADLIRDIDAMRWFLFEDTGV      383
Pa_SctV         GGGKPKAGRLAEQ----EEFALTVPLLIDVDASLQERLEAMSLNEELVRVRRALYLDFGV      409
Ye_SctV         SG--ANKGRLGEQ----EAFAMTVPLLIDVDSSQQEALEAIALNDELVRVRRALYLDLGV      407
Yp_SctV         SG--ANKGRLGEQ----EAFAMTVPLLIDVDSSQQEALEAIALNDELVRVRRALYLDLGV      407
                                      : **:: .       :.:  :   :  :*  :: * **

Sf_SctV         RLPTILYRTSNELKVDDIVLLINEVRADSFNIY--FDKVCITDENGDIDALGIPVVST--      450
S2_SctV         PLPEVNIEVLPEPTEKLTVLLYQE-PVFSLSIPAQADYLLIGADASVVG------DSQTL      436
Pa_SctV         PFPGIHLRFNEAMGDGEYLVQLQEVPVAR--GCLRPGWLLVRERAAQLELLAVPHEPAEL      467
Ye_SctV         PFPGIHLRFNEGMGEGEYLISLQEVPVAR--GELKAGYLLVRESVSQLELLGIPYEKGEH      465
Yp_SctV         PFPGIHLRFNEGMGEGEYIISLQEVPVAR--GELKAGYLLVRESVSQLELLGIPYEKGEH      465
                 :* :  .          ::  :*  .         . : :    . :

Sf_SctV         SYNERVISWVDVSYTENLTNIDAKIKSAQDEFYHQLSQALLNNINEIFGIQETKNMLDQF      510
S2_SctV         PNGMGQICWLTKDMAHKAQGFGLDVFAGSQRISALLKCVLLRHMGEFIGVQETRYLMNAM      496
Pa_SctV         QVPGEEASWVEQAHQDRLERSGCACLGLEQVLTWHLSHVLREYAEDFIGIQETRYLLEQM      527
Ye_SctV         LLPDQETFWVSVEYEERLEKSQLEFFSHSQVLTWHLSHVLREYAEDFIGIQETRYLLEQM      525
Yp_SctV         LLPDQEAFWVSVEYEERLEKSQLEFFSHSQVLTWHLSHVLREYAEDFIGIQETRYLLEQM      525
                        *:     ..         . .: :   *. .* .   :::*:***: ::: :
```

```
Sf_SctV      ENRYPDLLKEVFRHVTIQRISEVLQRLLGENISVRNLKLIMESLALWAPREKDVITLVEH      570
S2_SctV      EKNYSELVKELQRQLPINKIAETLQRLVSERVSIRDLRLIFGTLIDWAPREKDVLMLTEY      556
Pa_SctV      EQGYGELVKEAQRIVPLQRMTEILQRLVGEDISIRNMRAILEAMVEWGQKEKDVVQLTEY      587
Ye_SctV      EGGYGELIKEVQRIVPLQRMTEILQRLVGEDISIRNMRSILEAMVEWGQKEKDVVQLTEY      585
Yp_SctV      EGGYGELIKEVQRIVPLQRMTEILQRLVGEDISIRNMRSILEAMVEWGQKEKDVVQLTEY      585
             *  * :*:**  * : :::::* ****:.* :*:*::: *: ::  *. :****: *.*:

Sf_SctV      VRASLSRYICSKIAVSGEIKV-VMLSGYIEDAIRKGIRQTSGGSFLNMDIEVSDEVMETL      629
S2_SctV      VRIALRRHILRRLNPEGKPLPILRIGEGIENLVRESIRQTAMGTYTALSSRHKTQILQLI      616
Pa_SctV      IRSSLKRYICYKYSSGNNILPAYLLDQAVEEQIRGGIRQTSAGSYLALDPAITQAFLERV      647
Ye_SctV      IRSSLKRYICYKYANGNNILPAYLFDQEVEEKIRSRVRQTSAGSYLALDPAVTESLLEQV      645
Yp_SctV      IRSSLKRYICYKYANGNNILPAYLFDQEVEEKIRSGVRQTSAGSYLALEPAVTESLLEQV      645
             :* :* *:*  :    .:      :.  :*: :*  :***: *::  :.   .  .:: :

Sf_SctV      AHALRELRNAKKNFVLLVSVDIRRFVKRLIDNRFKSILVISYAEIDEAYTINVLKTI---      686
S2_SctV      EQAL----KQSAKLFIVTSVDTRRFLRKITEATLFDVPILSWQELGEESLIQVVESIDLS      672
Pa_SctV      RQTVGDLAQMQNRPVLIVSMDIRRYVRKLVESDYAGLPVLSYQELTQQINIQPLGRIVL-      706
Ye_SctV      RKTIGDLSQIQSKPVLIVSMDIRRYVRKLIESEYYGLPVLSYQELTQQINIQPLGRVCL-      704
Yp_SctV      RKTIGDLSQIQSKPVLIVSMDIRRYVRKLIESEYYGLPVLSYQELTQQINIQPLGRICL-      704
              :::    : . . .::.*:* **::::: :    .: ::*: *: :   *: :  :

Sf_SctV      ---------    686
S2_SctV      EEELADNEE    681
Pa_SctV      ---------    706
Ye_SctV      ---------    704
Yp_SctV      ---------    704
```

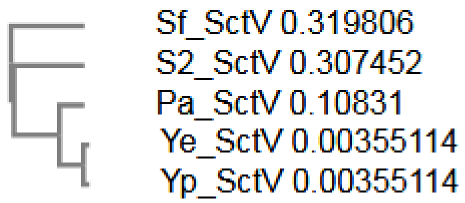

Phobius (http://phobius.sbc.su.se) topology prediction for Ye_SctV:

```
FT   TOPO_DOM      1     17       CYTOPLASMIC.
FT   TRANSMEM     18     36
FT   TOPO_DOM     37     41       NON CYTOPLASMIC.
FT   TRANSMEM     42     61
FT   TOPO_DOM     62     72       CYTOPLASMIC.
FT   TRANSMEM     73     93
FT   TOPO_DOM     94    104       NON CYTOPLASMIC.
FT   TRANSMEM    105    131
FT   TOPO_DOM    132    199       CYTOPLASMIC.
FT   TRANSMEM    200    220
FT   TOPO_DOM    221    231       NON CYTOPLASMIC.
FT   TRANSMEM    232    259
FT   TOPO_DOM    260    279       CYTOPLASMIC.
FT   TRANSMEM    280    298
FT   TOPO_DOM    299    303       NON CYTOPLASMIC.
FT   TRANSMEM    304    321
FT   TOPO_DOM    322    704       CYTOPLASMIC
```

SctK

```
CLUSTAL O(1.2.4) multiple sequence alignment

Sf_SctK      MIRMDGIYKKYLSIIFDPAFYINRNRLNLPSE----LLENGVIRSEINNLIINKYDLNCD      56
Ye_SctK      ---MMENYITSFQLRFCPAAYLHLEQLPSLWRSILPYLPQWRDSAHLNAALLDEFSLDTD      57
Yp_SctK      ----MENYITSFQLRFCPAAYLHLEQLPSLWRSILPYLPQWRDSAHLNAALLDEFSLDTD      56
Pa_SctK      ------MPLTAYQLRFCPARYIHESHLPAVLLRLLPALPDWRRQSVLNAWLLEQLELDCA      54
                      .  .: * ** *:: .:*          * :    : :*  :::: .*:

Sf_SctK      IEPLSGVTAMFVANWNLLPAVAYFIGSQESRLINHSEMVISYYGG--------KISKQGE      108
Ye_SctK      YEEPHGLGALPLQPQSQLELLLCRLGL-----VLHGEAIRRCVLASPLQQLLTLVNQETL      112
Yp_SctK      YEEPHGLGALPLQPQSQLELLLCRLGL-----VLHGEAIRRCVLASPLQQLLTLVNQETL      111
Pa_SctK      FRMPAQLGGLALYPQAALERTLGWLGA-----LLHGQALRQVLDGARVRRIRAQIGEQGQ      109
              .    : .: :     *      :*      : *.: :     .         :.::

Sf_SctK      A------AIRSGFWHLIAWK----ENISVGIYERINLLFNPIALEGNYTPVERNLS-RL-      156
Ye_SctK      RQIIVQHELLIGPWP-TNWQRPLPTEIESRTMIQSGLAFWLAAMEPQPQAWCKRLSLRLP      171
Yp_SctK      RQIIVQHELLIGPWP-THWQRPLPTEIESRTMIQSGLAFWLAAMEPQPQAWCKRLSLRLP      170
Pa_SctK      RFCLEQLDLLIGRWP-PGWQRALPENPEEGYFRRCGLAFWLAACSDADCGFSRRLRLRLR      168
                     :  * *    *:     : .     : .* *   * .       :.*  **

Sf_SctK      ------NEGMQYAKRHFTGIQTSCL---------------      175
Ye_SctK      LA--TPSEPWLVAESQRPLAQTLCHKLVKQVMPTCSHLFK      209
Yp_SctK      LA--TPSEPWLVAESQRPLAQTLCHKLVKQVTPTCSHLFK      208
Pa_SctK      LEAMPAPADWTFDEQRRSLARTLCLKVARQASDECFHLLN      208
                          : :    :* *
```

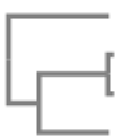

SctQ

CLUSTAL O(1.2.4) multiple sequence alignment

```
Sf_SctQ      ------------------------------------------------------------      0
S2_SctQ      ------------------MLRIANEERPWVEILPTQGATIGELTLSMQQYPVQQGTLFTI      42
Pa_SctQ      MNGADLDLPLASRAELDLQRRLARCRRHYVG-------NALQARLDIAQAAPDVDLELSL      53
Ye_SctQ      --MSLLTLPQAKLSELSLRQRLSHYRQNYLW-------EEGKLELTVSEPPSSLNCILQL      51
Yp_SctQ      --MSLLTLPQAKLSELSLRQRLSHYQQNYLW-------EEGKLELTVSEPPSSLNCILQL      51


Sf_SctQ      --------------MCGDWVIR-IDTLSFLKKKYEVFSGFSTQES--LLHL---SKCVFI      40
S2_SctQ      NYHNELGRVWIAEQCWQRWCEGLIGTANRSAIDPELLYGIAEWGLAPLLQASDATLCQNE      102
Pa_SctQ      AWDGLPLRFLCQAPALARWLAPNLQEAAFASLPAALQLALLEREGN----VFPGLVWYGL      109
Ye_SctQ      QWKGTHFTLYCFGDDLANWLTPDLLGAPFSTLPKELQLALLERQTV----FLPKLVCNDI      107
Yp_SctQ      QWKGTHFTLYCFGNDLANWLTADLLGAPFFTLPKELQLALLERQTV----FLPKLVCNDI      107
                               *    :           : .:

Sf_SctQ      ESSSVFSIPELSDKITFRITNEIQYATTGSHLCCFSSSLGIIYFDKMPVLRNQVSLDSLH      100
S2_SctQ      PPTSC---SNLPHQLALHIKWTVEEHEFHSIIFTWPTGFLRNIVGELSAERQQIYPAPP-      158
Pa_SctQ      SPAQP---RA-A--MGLRL--SLERD--DQRLALWLDGDPATLLARLPPRPSAQRLAIP-      158
Ye_SctQ      ATASL---SVTQ--PLLSL--RLSRD--NAHISFWLTSAE-ALFALLPARPNSERIPLP-      156
Yp_SctQ      ATASL---SVTQ--PLLSL--RLSRD--NAHISFWLTSAE-ALFALLPARPNSERIPLP-      156
               :.            : :   :.       :  :  .     .  :    .

Sf_SctQ      HLLEFCLGSSN--VRLATLKRIRTGDIIIVQKLYNLL--------------------LCN      138
S2_SctQ      VVVPVYSGWCQL--TLIELESIEIGMGVRIHCFGDIRLGFFAIQLPGGIYA---RVLLTE      213
Pa_SctQ      --LRLSLQWPGLPLDASELRTLEPGDLL--------------L-LPAGHRPDAALLGVLE      201
Ye_SctQ      --ILLSLRWHKVYLTLDEVDSLRLGDVL--------------L-APEGSGPNSPVLAYVG      199
Yp_SctQ      --ILISLRWHKVYLTLDEVDSLRLGDVL--------------L-APEGSGPNSPVLAYVG      199
               : .             :  :. *  :

Sf_SctQ      QVIIGDYIVNDNNEAKIN----LSESNGESEHTEVSLALFNYDDINVKVDFILLEKNMTI      194
S2_SctQ      DNTMKFDELVQDIETLLASGSPMSKSDGT--------SSVELEQIPQQVLFEVGRASLEI      265
Pa_SctQ      GRPWARCQLHSTQLELLDMHDTPSLADGE--------DLHELDQLPIPVSFEVGRRTLDL      253
Ye_SctQ      ENPWGYFQLQSNKLEFIGMSHESDELNPK--------PLTDLNQLPVQVSFEVGRQILDW      251
Yp_SctQ      ENPWGYFQLQSNKLEFIGMSHESDELNPE--------PLTDLNQLPVQVSFEVGRQILDW      251
                     : .     :      .  :             : :::   * * : .  :

Sf_SctQ      NELKMYVENELFKFPDDIVKHVNIKVNGSLVGHGELVSIEDGYGIEISSWMVKE---   248
S2_SctQ      GQLRQLKTGDVLPVGGCFAPEVTIRVNDRIIGQGELIACGNEFMVRITRWYLCKNTA   322
Pa_SctQ      HTLSTLQPGSLLDLDSALDGEVRILANQRCLGIGELVRLQDRLGVRVTRLFGHDEA-   309
Ye_SctQ      HTLTSLEPGSLIDLTTPVDGEVRLLANGRLLGHGRLVEIQGRLGVRIERLTEVTIS-   307
Yp_SctQ      HTLTSLEPGSLIDLTTPVDGEVRLLANGRLLGHGRLVEIQGRLGVRIERLTEVTIS-   307
               *     ..::  .   .  .* : .*   :* *.*:   .   :.:
```

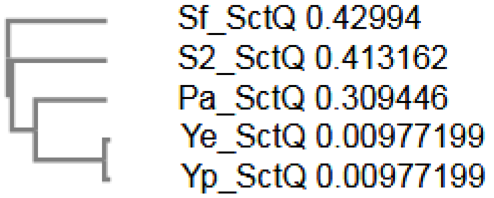

SctL

```
CLUSTAL O(1.2.4) multiple sequence alignment

Sf_SctL      MKVCNMQKGTLPVSRHHAYDGVVIKRIEKEL-----CKTIKDRDTESKKKAICVIKEATK      55
S2_SctL      -----MSFTSLPLTEIN-HKLPARNIIESQW-ITL-QLTLFAQEQQAKRVSHAIVSSAYR      52
Pa_SctL      -------------------MLPFVELDASRVRLAPGQALLRARDYQDYLSANRLVEAARE      41
Ye_SctL      -----MSQTC--QTGYA-YMQPFVQIIPSNLSLACGLRILRAEDYQSSLTTEELISAAKQ      52
Yp_SctL      -------------------MQPFVQIIPSNLSLACGLRILRAEDYQSSLTTEELISAAKQ      41
                                     :   ..         :  .: :    :  ::. * .

Sf_SctL      KAESLRIDAV-------CDGYQIGIQTAF---EHIIDYICEWKLKQNENRRNIEDYITSL      105
S2_SctL      KAEKIIRDAYRYQREQKV-EQQQ--ELACLRKNTLEKMEVEWLEQHVKHLQDDENQFRSL      109
Pa_SctL      RAAEIEREAHEVYQEQKRLGWEAGLEEARLRQAGLIQETL---LRCNRYYRQVDRQLGEV      98
Ye_SctL      DAEKILADAQEVYEQQKQLGWQAGMDEARTLQATLIHETQ---LQCQQFYRHVEQQMSEV      109
Yp_SctL      DAEKILADAQEVYEQQKQLGWQAGMDEARTLQATLIHETQ---LQCQQFYRHVEQQMSEV      98
              * .:  :*            :   : *      : .       :  .  :. :  : .:

Sf_SctL      LSENLHD-ERIISTLLEQWLSSLRNTV-TELK-----------------VVLPKCNLALR      146
S2_SctL      VDHAAHHIKNSIEQVLLAWFDQQSVDSVMCHRLARQAT-AMAEEGALYLRIHPEKEALMR      168
Pa_SctL      VLQAVRKVLRH--------YDAVEL----TLAATREALALVSNQKQVILHVQPEQLAAVR      146
Ye_SctL      VLLAVRKILND--------YDQVDM----TLQVVREALALVSNQKQVVVRVNPDQAGTIR      157
Yp_SctL      VLLAVRKILND--------YDQVAM----TLQVVREALALVSNQKQVVVRVNPDQAGAIR      146
             :    :.  .          .                             : *.    :*

Sf_SctL      KKLELDLHKYRSDVKIILKYSE--------GNNYIFCSGNQVVEFSPQDVISGVKIELAE      198
S2_SctL      ETFGKRFTLI-----IEPGFSPDQAE---------LSSTRYAVEFSLSRHFNALLKWL--      212
Pa_SctL      EQVARVLKDF-----PEVGYLEVVGDARLDQGGCILETEIGIIDASLDSQLAALQAALTE      201
Ye_SctL      EQIAKVHKDF-----PEISYLEVTADARLDQGGCILETEVGIIDASIDGQIEALSRAIST      212
Yp_SctL      EQIAKVHKDF-----PEISYLEVTADARLDQGGCILETEVGIIDASIDGQIEALSRAIST      201
             : .                :               : :    :: * .  : .:   :

Sf_SctL      KLTK--------NDKKYFKELAHKKLRQIAEDLLKENPVND     231
S2_SctL      -----RNGEDKRGSDEY------------------------     224
Pa_SctL      SVARSGEEEGDAG----------------------------     214
Ye_SctL      TLGQMKVTEEE------------------------------     223
Yp_SctL      TLGQMKVTE--------------------------------     210
```

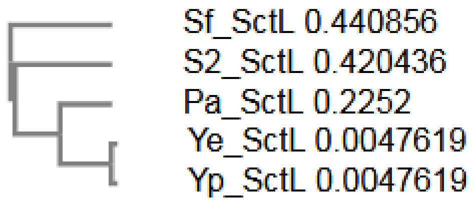

SctN

```
CLUSTAL O(1.2.4) multiple sequence alignment

Sf_SctN      ---------------MSYTKLLTQLSFPNRISGPILETSLSDVSIGEICNIQAGIESNEI      45
S2_SctN      ---MKNELMQRLRLKYPPPDGYCRWGRIQDVSATLLNAWLPGVFMGELCCIKPGEE----      53
Pa_SctN      MPAPLSPLIVRMRHAIEGCRPIQIRGRVTQVTGTLLKAVVPGVRIGELCQLRNPDQSL--      58
Ye_SctN      -MLSLDQIPHHIRHGIVGSRLIQIRGRVTQVTGTLLKAVVPGVRIGELCYLRNPDNSL--      57
Yp_SctN      -MLSLDQIPHHIRHGIVGSRLIQIRGRVTQVTGTLLKAVVPGVRIGELCYLRNPDNSL--      57
                                      .    ::. :*:: : .* :**:* ::   :

Sf_SctN      VARAQVVGFHDEKTILSLIGNSRGLSRQTLIKPTAQFLHTQVGRGLLGAVVNPLGEVTDK      105
S2_SctN      --LAEVVGINGSKALLSPFTSTIGLHCGQQVMALRRRHQVPVGEALLGRVIDGFGRPLDG      111
Pa_SctN      ALLAEVIGFQQHQALLTPLGEMLGVSSNTEVSPTGGMHRVAVGEHLLGQVLDGLGRPFDG      118
Ye_SctN      SLQAEVIGFAQHQALLIPLGEMYGISSNTEVSPTGTMHQVGVGEHLLGQVLDGLGQPFDG      117
Yp_SctN      SLQAEVIGFAQHQALLIPLGEMYGISSNTEVSPTGTMHQVGVGEHLLGQVLDGLGQPFDG      117
                *:*:*:   :::*  : .  *:     :       :. **. *** *:: :*.  *

Sf_SctN      FAVTDNSEILYRPVDNAPPLYSERAAIEKPFLTGIKVIDSLLTCGEGQRMGIFASAGCGK      165
S2_SctN      RELPD---VCWKDYDAMPPPAMVRQPITQPLMTGIRAIDSVATCGEGQRVGIFSAPGVGK      168
Pa_SctN      SPPAE--PAAWYPVYRDAPQPMSRRLIERPLSLGVRAIDGLLTCGEGQRMGIFAAAGGGK      176
Ye_SctN      GHLPE--PAAWYPVYQDAPAPMSRKLITTPLSLGIRVIDGLLTCGEGQRMGIFAAAGGGK      175
Yp_SctN      GHLPE--PAAWYPVYQDAPAPMSRKLITTPLSLGIRVIDGLLTCGEGQRMGIFAAAGGGK      175
                 :     :       *    *  *  *:  *::.**.: *******:***:: * **

Sf_SctN      TFLMNMLIEHSGADIYVIGLIGERGREVTETVDYLKNSEKKSRCVLVYATSDYSSVDRCN      225
S2_SctN      STLLAMLCNAPDADSNVLVLIGERGREVREFIDFTLSEETRKRCVIVVATSDRPALERVR      228
Pa_SctN      STLLASLVRNAEVDVTVLALVGERGREVREFIESDLGEQGLRRSVLVVATSDRPAMERAK      236
Ye_SctN      STLLASLIRSAEVDVTVLALIGERGREVREFIESDLGEEGLRKAVLVVATSDRPSMERAK      235
Yp_SctN      STLLASLIRSAEVDVTVLALIGERGREVREFIESDLGEEGLRKAVLVVATSDRPSMERAK      235
             : *:  * .   .*  *: *:******* * ::   ..:   :.*:* ****  :::* .

Sf_SctN      AAYIATAIAEFFRTEGHKVALFIDSLTRYARALRDVALAAGESPARRGYPVSVFDSLPRL      285
S2_SctN      ALFVATTIAEFFRDNGKRVVLLADSLTRYARAAREIALAAGETAVSGEYPPGVFSALPRL      288
Pa_SctN      AGFVATSIAEYFRDQGRRVLLLMDSLTRFARAQREIGLAAGEPPTRRGYPPSVFAALPRL      296
Ye_SctN      AGFVATSIAEYFRDQGKRVLLLMDSVTRFARAQREIGLAAGEPPTRRGYPPSVFAALPRL      295
Yp_SctN      AGFVATSIAEYFRDQGKRVLLLMDSVTRFARAQREIGLAAGEPPTRRGYPPSVFAALPRL      295
             * ::**:***:** :*::* *: **:**:*** *::.*****  .   ** .** :****

Sf_SctN      LERPGKLKAGGSITAFYTVLLEDDDFADPLAEEVRSILDGHIYLSRNLAQKGQFPAIDSL      345
S2_SctN      LERTGMG-EKGSITAFYTVLVEGDDMNEPLADEVRSLLDGHIVLSRRLAERGHYPAIDVL      347
Pa_SctN      MERAGQS-ERGSITALYTVLVEGDDMSEPVADETRSILDGHIVLSRKLAAANHYPAIDVL      355
Ye_SctN      MERAGQS-SKGSITALYTVLVEGDDMTEPVADETRSILDGHIILSRKLAAANHYPAIDVL      354
Yp_SctN      MERAGQS-SKGSITALYTVLVEGDDMTEPVADETRSILDGHIILSRKLAAANHYPAIDVL      354
             :** *     *****:****:*.**: :*:*:*.**:***** ***.**  .::**** *

Sf_SctN      KSISRVFTQVVDEKHRIMAAAFRELLSEIEELRTIIDFGEYKPGENASQDKIYNKISVVE      405
S2_SctN      ATLSRVFPVVTSHEHRQLAAILRRCLALYQEVELLIRIGEYQRGVDTDTDKAIDTYPDIC      407
Pa_SctN      HSVSRVMNQIVDDDQRHAAGRLREWLAKYEEVELLLKIGEYQKGQDSEADRAIEKIGAIR      415
Ye_SctN      RSASRVMNQIVSKEHKTWAGDLRRLLAKYEEVELLLQIGEYQKGQDKEADQAIERIGAIR      414
Yp_SctN      RSASRVMNQIVSKEHKTWAGDLRRLLAKYEEVELLLQIGEYQKGQDKEADQAIERMGAIR      414
              : ***:  :....::  *. :*. *:  :*:. :: :***: * : . *:  :    :

Sf_SctN      SFLKQDYRLGFTYEQTMELIGETIR- 430
S2_SctN      TFLRQSKDEVCGPELLIEKLHQILTE 433
Pa_SctN      QWLRQGTHETSDYAQACAQLRSLCA- 440
Ye_SctN      GWLCQGTHELSHFNETLNLLETLTQ- 439
Yp_SctN      GWLCQGTHELSHFNETLNLLETLTQ- 439
              :* *.              :
```

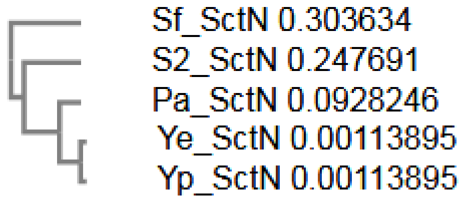

Supplementary Table 1 – Multiple sequence alignment of T3SS components

Multiple sequence alignments for transmembrane and cytosolic T3SS components analyzed in this study (SctC, D, J, V, K, Q, L, N) from different species (*Y. enterocolitica, Y. pestis, P. aeruginosa, S. flexneri* and *S. enterica S*PI-2). (**a**) Overview of protein similarity. Identical and similar amino acids were determined by a BlastP pairwise protein alignment (https://blast.ncbi.nlm.nih.gov) of the given proteins with the *Y. enterocolitica* W22703 pYVe227 homolog, using standard settings, only displaying best homology stretches with E values < 0.1. *: no clearly homologous protein known; n.d.: no homology stretch with E < 0.1 detected. (**b**) Multiple sequence alignment. Sequence alignment was performed using Clustal Omega 1.2.4 (https://www.ebi.ac.uk/Tools/msa/clustalo/) [4] using NCBI protein sequences for the strains listed in (A). Phylograms (Clustal Omega Guide Tree, standard settings, real branch length) are displayed below the individual alignments. For SctV, the result of a Phobius topology prediction (http://phobius.sbc.su.se/) [3] is displayed for *Y. enterocolitica*. For amino acids predicted to be located in the periplasm or extracellular space, His residues in *Y. enterocolitica* are marked in red. Neutral amino acids at the equivalent position in *P. aeruginosa* are marked in blue. For none of these amino acids, the aligned His residue was conserved for *S. flexneri*. Val-632 in *S*PI-2 SctV [5] is marked in green.

Strain	Genotype	Reference
MRS40	Wild-type pYV *Y. enterocolitica* E40 *ΔblaA*	[6]
IML421*asd* (HOPEMTasd)	MRS40 *yopO*$_{\Delta 12\text{-}427}$ *yopE*$_{21}$ *yopH*$_{\Delta 11\text{-}352}$ *yopM*$_{23}$ *yopP*$_{23}$ *yopT*$_{135}$ *Δasd*	[7]
AD4016	MRS40 *egfp-sctQ*	[8]
AD4085	IML421*asd egfp-sctQ*	[7]
AD4175	IML421*asd sctV-egfp* (mutated with pAD208)	This work
AD4306	IML421*asd egfp-sctD*	[9]
AD4411	IML421*asd egfp-sctQ ΔsctD*	[10]
AD4439	IML421*asd pamcherry1-sctD* (mutated with pAD439)	This work
AD4474	IML421*asd efgp-sctK*	[10]
ADTM4514	IML421*asd egfp-sctN*	[10]
ADTM4520	IML421*asd egfp-sctL*	[10]
ADTM4521	IML421*asd mcherry-sctL*	[10]
ADTM4525	IML421*asd halo-sctL*	[10]
ADMH4536	IML421*asd halo-sctL ΔsctF*	This work
DL001	*P. aeruginosa* PAO1 *egfp-sctQ*	[11]
S. flexneri gfp-sctN	*S. flexneri* serotype 2a 2457T *gfp-sctN(spa47)*	[12]

Plasmids	Genotype	Reference
pBAD-His B	pBR322-derived expression vector	Invitrogen
pKNG101	*oriR6K sacBR+ oriTRK2 strAB+* (suicide vector)	[13]
pAD208	*pKNG101-sctV-egfp*	[10]
pAD439	*pKNG101-pamcherry1-sctD*	This work
pAD141	*pBAD::sctJ*	This work
pAD476	*pBAD::egfp*	[10]
pAD477	*pBAD::mCherry*	[10]
pAD603	*pBAD::sctV*	This work
pAD638	*pBAD::sctF*$_{S5C}$	This work
pEE010	*pBAD::sctD*$_{H193A,H205S,R214H,H353Y,H376G}$	This work
pISO85	*pKNG101-ΔsctF*	[8]
pSW001	*pBAD::pHluorin*	This work
pSW022	*pBAD::sctD*	This work
pSW023	*pBAD::sctC*	This work

Supplementary Table 2 – Strains and plasmids used in this study

Except for pSW044, all plasmids encoding T3SS components use the *Yersinia enterocolitica* variant.

**Low salt LB medium**	**concentration**
Yeast extract	5 g/l
Trypton	10 g/l
NaCl	3 g/l
Agarose (for plates)	1.5%

**BHI medium**	**concentration**
Brain heart infusion solids	17.5 g/l
Peptones	10.0 g/l
Glucose	2.0 g/l
Sodium chloride	5.0 g/l
Disodium hydrogen phosphate	2.5 g/l
Agarose (for plates)	1.5%

**Minimal microcopy medium**	**concentration**
HEPES pH 7.2	100 mM
$(NH_4)_2SO_4$, ammonium sulfate	5 mM
NaCl	100 mM
Sodium glutamate	20 mM
$MgCl_2$	10 mM
K_2SO_4	5 mM
MES	5 mM
Glycine	50mM
Casamino acids	0.5%
Agarose (for agarose pads)	1.5%

Supplementary Table 3 – Composition of media used in this study

Supplementary references

1. Yu, X.-J., Liu, M., Matthews, S. & Holden, D. W. Tandem translation generates a chaperone for the Salmonella type III secretion system protein SsaQ. *J. Biol. Chem.* **286**, 36098–107 (2011).
2. Bzymek, K. P., Hamaoka, B. Y. & Ghosh, P. Two translation products of Yersinia yscQ assemble to form a complex essential to type III secretion. *Biochemistry* **51**, 1669–77 (2012).
3. Käll, L., Krogh, A. & Sonnhammer, E. L. L. Advantages of combined transmembrane topology and signal peptide prediction--the Phobius web server. *Nucleic Acids Res.* **35**, W429-32 (2007).
4. Sievers, F. *et al.* Fast, scalable generation of high-quality protein multiple sequence alignments using Clustal Omega. *Mol. Syst. Biol.* **7**, 539 (2011).
5. Yu, X.-J., Grabe, G. J., Liu, M., Mota, L. J. & Holden, D. W. SsaV Interacts with SsaL to Control the Translocon-to-Effector Switch in the Salmonella SPI-2 Type Three Secretion System. *MBio* **9**, e01149-18 (2018).
6. Sory, M.-P., Boland, A., Lambermont, I. & Cornelis, G. R. Identification of the YopE and YopH domains required for secretion and internalization into the cytosol of macrophages, using the cyaA gene fusion approach. *Proc. Natl. Acad. Sci. U. S. A.* **92**, 11998–12002 (1995).
7. Kudryashev, M. *et al.* In situ structural analysis of the Yersinia enterocolitica injectisome. *Elife* **2**, e00792 (2013).
8. Diepold, A. *et al.* Deciphering the assembly of the Yersinia type III secretion injectisome. *EMBO J.* **29**, 1928–40 (2010).
9. Diepold, A., Kudryashev, M., Delalez, N. J., Berry, R. M. & Armitage, J. P. Composition, Formation, and Regulation of the Cytosolic C-ring, a Dynamic Component of the Type III Secretion Injectisome. *PLOS Biol.* **13**, e1002039 (2015).
10. Diepold, A. *et al.* A dynamic and adaptive network of cytosolic interactions governs protein export by the T3SS injectisome. *Nat. Commun.* **8**, 15940 (2017).
11. Lampaki, D., Diepold, A. & Glatter, T. A Serial Sample Processing Strategy with Improved Performance for in-Depth Quantitative Analysis of Type III Secretion Events in Pseudomonas aeruginosa. *J. Proteome Res.* **19**, 543–553 (2020).
12. Burgess, J. L., Case, H. B., Burgess, R. A. & Dickenson, N. E. Dominant negative effects by inactive Spa47 mutants inhibit T3SS function and Shigella virulence. *PLoS One* **15**, e0228227 (2020).
13. Kaniga, K., Delor, I. & Cornelis, G. R. A wide-host-range suicide vector for improving reverse genetics in Gram-negative bacteria: inactivation of the blaA gene of Yersinia enterocolitica. *Gene* **109**, 137–41 (1991).
