# Supplementary material for: Dynamic relocalization of cytosolic type III secretion system components prevents premature protein secretion at low external pH
Source: Nat Commun. 2021 Mar 12;12:1625. doi: 10.1038/s41467-021-21863-4 (PMC7954860; doi:10.1038/s41467-021-21863-4)
Supplement: Supplementary file 3 — Descriptions of Additional Supplementary Files [file 41467_2021_21863_MOESM3_ESM.pdf]

Descriptions of Additional Supplementary Files

Supplementary Data 1

Description: Distribution of number of EGFP-SctQ fluorescent foci over time upon change of external pH Number of bacteria with n (blue font, left) detected spots over time (in seconds, top row); relates to Suppl. Fig. 9. (A) Dissociation of foci upon shift of external pH from 7 to 4. (B) Reassociation of foci upon shift of external pH from 4 to 7. Spot numbers per bacterium were determined using the customized BiofilmQ software for 337-349 bacteria (top) / 328-347 bacteria (bottom, except for t=40 s, where n=240 detected bacteria) from three independent experiments in each case. Please refer to Source data file for number of bacteria at individual time points. Darker colors identify combinations with a higher number of bacteria.

Supplementary Movie 1

Description: Time-lapse phase contrast video of Y. enterocolitica attached to a glass cover slip in a flow cell at pH 7. The buffer was exchanged from pH 7 to pH 4 buffered media during the experiment and cells were tracked for 10 minutes with a picture taken every 10 seconds.

Supplementary Movie 2

Description: Time-lapse phase contrast video of Y. enterocolitica attached to a glass cover slip in a flow cell at pH 4. During the experiment the buffer was changed from pH 4 to pH 7 and cells were tracked again for 10 minutes with a picture taken every 10 seconds. Scale bars, 2 µm.

Supplementary Movie 3

Description: The pH-induced dissociation and reassociation of EGFP-SctK to the injectisome can be repeated for several cycles Time-lapse video of Y. enterocolitica expressing EGFP-SctK attached to a glass cover slip in a flow cell. After flow was started the buffer was toggled every 5 minutes between pH 7 to pH 4, as indicated. Micrographs were acquired every 10 seconds. Scale bar, 2 µm.

Supplementary Movie 4

Description: Dissociation kinetics of EGFP-SctQ upon change of external pH from 7 to 4 Time-lapse video of Y. enterocolitica expressing EGFP-SctQ attached to a glass cover slip in a flow cell after pH shift from 7 to 4. Left, overlay of DIC (grey) and fluorescence signal (yellow); right, fluorescent channel in red hot color scale. Bacteria were attached to the cover slip at pH 7, flow was introduced, and the buffer was switched from pH 7 to pH 4. The duration of the experiment was 10 minutes and pictures were taken every 10 seconds. Scale bar, 2 µm.

Supplementary Movie 5

Description: Re-association kinetics of EGFP-SctQ upon change of external pH from 4 to 7 Time-lapse video of Y. enterocolitica expressing EGFP-SctQ attached to a glass cover slip in a flow cell after pH shift from 4 to 7. Left, overlay of DIC (grey) and fluorescence signal (yellow); right, fluorescent channel in red hot color scale. Bacteria were attached to the cover slip at pH 7, flow was introduced,

and the buffer was switched from pH 4 to pH 7. The duration of the experiment was 10 minutes and pictures were taken every 10 seconds. Scale bar, 2 µm
